# Genome-wide association study identifies three novel loci in Fuchs endothelial corneal dystrophy

**DOI:** 10.1038/ncomms14898

**Published:** 2017-03-30

**Authors:** Natalie A. Afshari, Robert P. Igo, Nathan J. Morris, Dwight Stambolian, Shiwani Sharma, V. Lakshmi Pulagam, Steven Dunn, John F. Stamler, Barbara J. Truitt, Jacqueline Rimmler, Abraham Kuot, Christopher R. Croasdale, Xuejun Qin, Kathryn P. Burdon, S. Amer Riazuddin, Richard Mills, Sonja Klebe, Mollie A. Minear, Jiagang Zhao, Elmer Balajonda, George O. Rosenwasser, Keith H Baratz, V. Vinod Mootha, Sanjay V. Patel, Simon G. Gregory, Joan E. Bailey-Wilson, Marianne O. Price, Francis W. Price, Jamie E. Craig, John H. Fingert, John D. Gottsch, Anthony J. Aldave, Gordon K. Klintworth, Jonathan H. Lass, Yi-Ju Li, Sudha K. Iyengar

**Affiliations:** 1 Shiley Eye Institute, University of California, La Jolla, California 92093, USA; 2 Department of Epidemiology and Biostatistics, Case Western Reserve University, Cleveland, Ohio 44106, USA; 3 Department of Ophthalmology, University of Pennsylvania, Philadelphia, Pennsylvania 19104, USA; 4 Department of Ophthalmology, Flinders Medical Centre, Flinders University, Adelaide, South Australia 5042, Australia; 5 Michigan Cornea Consultants, PC, Southfield, Michigan 48034, USA; 6 Department of Ophthalmology, University of Iowa, College of Medicine, Iowa City, Iowa 52242, USA; 7 Duke Molecular Physiology Institute, Duke University Medical Center, Durham, North Carolina 27701, USA; 8 Davis Duehr Dean Clinic, Madison, Wisconsin 53715, USA; 9 Menzies Institute for Medical Research, University of Tasmania, Hobart, Tasmania 7000, Australia; 10 The Wilmer Eye Institute, Johns Hopkins University School of Medicine, Baltimore, Maryland 21287, USA; 11 Department of Pathology, Flinders Medical Centre, Flinders University, Adelaide, South Australia 5042, Australia; 12 Duke University Eye Center, Duke University Medical Center, Durham, North Carolina 27710, USA; 13 Central Pennsylvania Eye Institute, Hershey, Pennsylvania 17033, USA; 14 Department of Ophthalmology, Mayo Clinic, Rochester, Minnesota 55905, USA; 15 Department of Ophthalmology, University of Texas Southwestern Medical Center, Dallas, Texas 75235, USA; 16 Computational and Statistical Genomics Branch, National Human Genome Research Institute, National Institutes of Health and Johns Hopkins University, Baltimore, Maryland 21224, USA; 17 Price Vision Group, Indianapolis, Indiana 46260, USA; 18 Stein Eye Institute, University of California, Los Angeles, California 90095, USA; 19 Department of Pathology, Duke University Medical Center, Durham, North Carolina 27710, USA; 20 Department of Ophthalmology and Visual Sciences, Case Western Reserve University and University Hospitals Eye Institute, Cleveland, Ohio 44106, USA; 21 Department of Biostatistics and Bioinformatics, Duke University Medical Center, Durham, North Carolina 27710, USA

## Abstract

The structure of the cornea is vital to its transparency, and dystrophies that disrupt corneal organization are highly heritable. To understand the genetic aetiology of Fuchs endothelial corneal dystrophy (FECD), the most prevalent corneal disorder requiring transplantation, we conducted a genome-wide association study (GWAS) on 1,404 FECD cases and 2,564 controls of European ancestry, followed by replication and meta-analysis, for a total of 2,075 cases and 3,342 controls. We identify three novel loci meeting genome-wide significance (*P*<5 × 10^−8^): *KANK4* rs79742895, *LAMC1* rs3768617 and *LINC00970/ATP1B1* rs1200114. We also observe an overwhelming effect of the established *TCF4* locus. Interestingly, we detect differential sex-specific association at *LAMC1*, with greater risk in women, and *TCF4*, with greater risk in men. Combining GWAS results with biological evidence we expand the knowledge of common FECD loci from one to four, and provide a deeper understanding of the underlying pathogenic basis of FECD.

## Abstract (web summary)

Fuchs endothelial corneal dystrophy (FECD) is one of the most common reasons for corneal transplantation, and is known to cluster in families. Here, the authors discover new genetic loci associated with FECD with sex-specific effects and implications for disease mechanism.

Corneal diseases are one of the most common causes of visual impairment and blindness worldwide, with Fuchs endothelial corneal dystrophy (FECD) being the most common indication for corneal transplantation in the United States^1^. FECD is a bilateral, slowly progressive disorder of the corneal endothelium affecting 4% of population above the age of 40 years in the United States^2^,^3^. This debilitating disorder becomes symptomatic in the fifth or sixth decade of life^4^,^5^ and affects two to four times more women than men^3^,^6^. Clinical presentation of FECD varies, but often includes corneal oedema that results in a loss of corneal clarity, painful episodes of recurrent corneal erosions and severely impaired visual acuity. Medical management is often inadequate requiring corneal transplantation. Histopathological changes in FECD include loss of endothelial cells, thickening of Descemet membrane (DM) due to excessive accumulation of extracellular matrix, formation of central excrescences on DM (corneal guttae) and subepithelial bullae and scarring^7^. FECD likely arises during neural crest differentiation^8^, but the reason for loss of functioning endothelial cells is unknown.

FECD often clusters within families, with heritability estimated at ∼39% (ref. 9). The understanding of genetics of FECD initially focussed on genetic linkage, as a consequence of the observation by Krachmer *et al*.^5^ that this disease showed a dominant mode of inheritance in families. Linkage and fine-mapping studies in families identified rare point mutations in *COL8A2* (ref. 10), *SLC4A11* (ref. 11) and *TCF8* (ref. 12); these constituted the early genetic discoveries of Fuchs dystrophy. Most recently, a genome-wide association study (GWAS) including 280 cases and 410 controls in both discovery and replication samples found common genetic variation in *TCF4* to be associated with Fuchs dystrophy, along with two other loci that did not reach genome-wide significance^13^. The association with *TCF4* has been replicated by multiple investigators, including our group^14^,^15^, and a subsequent publication suggested that a trinucleotide repeat expansion within intron 3 of the *TCF4* gene is risk factor for this disease^16^. Here, we report the discovery of three new loci associated with FECD, *KANK4* (KN motif- and ankyrin repeat domain-containing protein 4), *LAMC1* (laminin gamma-1) and *LINC009970/ATPB1* (Na^+^, K^+^ transporting ATPase, beta-1 polypeptide), and confirm the strong effect of *TCF4* variants in our large samples of FECD cases and controls. Moreover, we show evidence for sex-specific effects for associated markers at *TCF4* and *LAMC1*, and describe the expression of the four risk loci in ocular tissues. The loci identified in this GWAS have not previously been reported for any other corneal dystrophy. Their discovery warrants further investigation into disease-causing mechanisms, including the maintenance of corneal endothelium integrity and fluid transportation.

## Results

### GWAS and replication of associated regions

**Figure 1:**
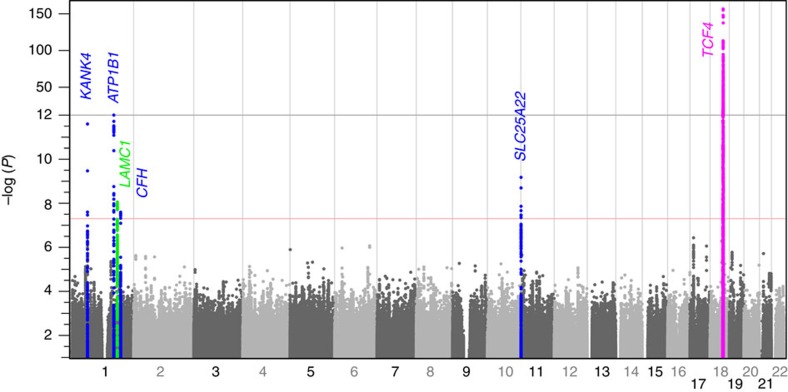
Results from GWAS for FECD. Negative log *P* values (vertical axis) from 8,680,745 SNPs are shown. The red horizontal line marks genome-wide significance (*P*=5 × 10^−8^). Negative log *P* values are condensed above *P*=10^−12^. Vertical grey lines are chromosome boundaries. The genomic control parameter^56^ was 1.033, indicating adequate control of test statistic inflation from population stratification (Supplementary Fig. 2).

GWAS was performed on a discovery data set of 3,968 unrelated subjects, which include 1,404 cases and 685 controls selected from two FECD genetic study groups^9^,^15^ and 1,879 controls from the Age-Related Eye Disease Study (AREDS) Refractive Error Substudy^17^ (Supplementary Tables 1–3). We identified six regions containing single-nucleotide polymorphisms (SNPs) with genome-wide significant associations (*P*<5 × 10^−8^ from logistic regression), of which 18 SNPs were advanced to replication (Fig. 1, Table 1 and Supplementary Figs 1 and 2). We also observed modest evidence for association at *AGBL1* (*P*=7.5 × 10^−5^) and *LOXHD1* (*P*=3.4 × 10^−4^), two of seven genes previously implicated in FECD through family-based and gene-targeted studies (Supplementary Table 4).

Three independent data sets with a total of 671 FECD cases and 778 controls served as replication (Supplementary Table 5). Four out of six regions showed strong evidence (*P*<5 × 10^−8^ from meta-analysis) for association with FECD in the discovery and replication cohorts (Table 1, Supplementary Table 6 and Supplementary Fig. 3). The previously identified locus at *TCF4* (refs 13, 14, 15, 18, 19, 20), encoding the transcription factor E2-2, remained the strongest association in both discovery and replication cohorts (most significant SNP rs784257; meta-*P*=2.5 × 10^−200^), with the largest effect sizes in both the discovery (odds ratio (OR)=5.77, 95% confidence interval (CI)=5.05, 6.60) and replication (OR=3.89, 95% CI=3.30, 4.59) samples. Replication was also successful in three regions on chromosome 1: intronic within the *KANK4* gene (meta-*P*=1.2 × 10^−14^ at rs79742895), intergenic region between *LINC00970* and the *ATP1B1* gene (*LINC00970*|*ATP1B1*, meta-*P*=9.9 × 10^−19^ at rs1022114) and intronic within the *LAMC1* gene (meta-*P*=6.9 × 10^−16^ at rs3768617). The discovery sample was well powered (>0.9) to detect association of the index markers at these four loci with the observed effect sizes (Supplementary Table 7).

### Properties of index variants

The most significant *TCF4* SNP, rs784257, explained 21.9% of the variation in FECD in the discovery sample, whereas the top markers in the other three replicated genome-wide significant loci each explained between 0.9% and 1.5% of the variation in the discovery sample (Supplementary Table 8). Strong linkage disequilibrium (*r*^2^=0.88) exists between rs784257 and the previously reported variant rs613872, in the third intron of *TCF4* (ref. 13). Both SNPs are in moderate disequilibrium with the expanded form of the CTG18.1 trinucleotide repeat in intron 2 of *TCF4* (*r*^2^=0.47), consistent with the previously reported values of 0.65 and 0.31 between the triplet repeat and rs613872 in FECD cases and controls, respectively^21^. The CTG18.1 repeat expansion is a strongly influential variant with good biologic plausibility^22^,^23^, but does not fully explain disease occurrence in all patients because of the lack of penetrance in some families^21^.

**Figure 2:**
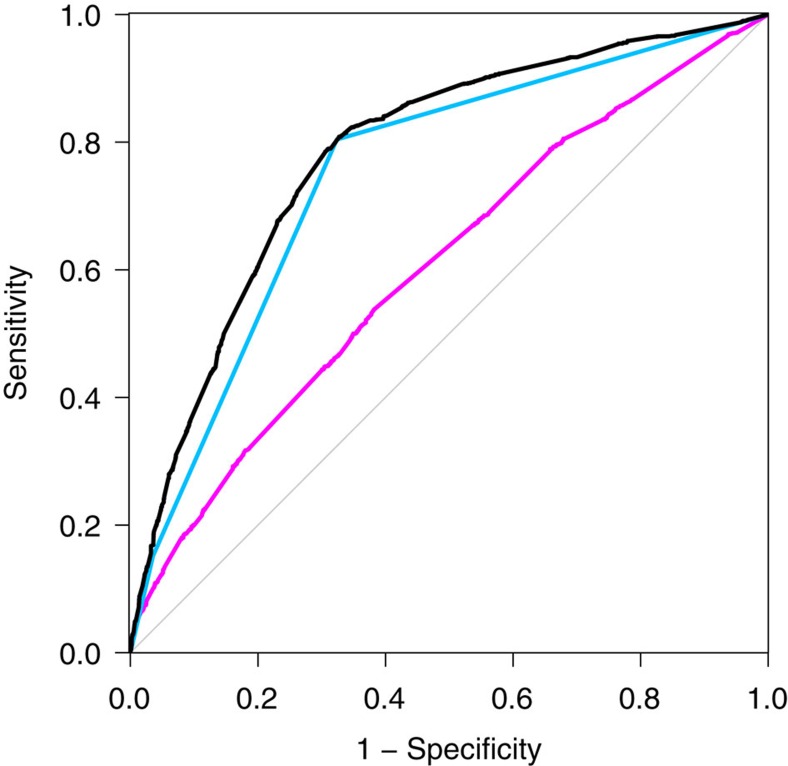
ROC curves for FECD status. Curves are shown from genetic risk scores based on all replicated loci (black), rs784257 in *TCF4* only (blue) and all loci except rs784257 (magenta).

Genetic risk scores were calculated for the discovery cohort based on the most significantly associated SNP from each of the four replicated regions. The area under the curve (AUC) for FECD cases versus controls was 0.782 (95% CI=0.767, 0.797), showing strong predictive value compared to other complex traits analysed by GWAS^24^ (Fig. 2). Predictive value was primarily through genotypes at rs784257 in *TCF4* (AUC=0.750; 95% CI=0.736–0.765); the AUC without rs784257 was substantially smaller but still significantly >0.5 (AUC=0.606; 95% CI=0.587–0.624). We also found that adding the three non-*TCF4* SNPs to the model significantly increased the AUC (*P*=7.7 × 10^−18^ by DeLong's test^25^).

Pairwise SNP × SNP interaction tests among the markers listed in Table 1 revealed no significant (*P*<0.05 from logistic regression including an SNP × SNP interaction term) interactions between loci. The discovery data set was large enough to detect an interaction risk ratio of 1.4–1.8 with at least 80% power (Supplementary Table 9). Conditional analysis on the *TCF4* marker rs784257 did not substantially alter the effect sizes of the other loci (Supplementary Table 10). Moreover, a sensitivity analysis using only FECD cases from the discovery cohort confirmed by histopathology (Supplementary Table 11) showed no overall reduction in effect sizes (Supplementary Table 12 and Supplementary Fig. 4), indicating that our classification of affected status is reliable.

From the sex-stratified analysis on the discovery cohort, we identified that the risk-associated major allele G of *LAMC1* variant rs3768617 confers a significantly greater elevated risk of FECD on women (OR=1.52, 95% CI=1.32, 1.72) than on men (OR=1.16, 95% CI=0.98, 1.34) (*P* value for heterogeneity, *P*_het_=0.013 by *χ*^2^ test; Supplementary Table 13 and Supplementary Figs 5 and 6), explaining 1.74% of the overall FECD risk for women in the sample, but only 0.23% of the risk for men. On the other hand, the *TCF4* variant rs784257 shows higher risk of FECD on men (OR=7.56, 95% CI=5.96, 9.57) than women (OR=5.06, 95% CI=4.29, 5.96, *P*_het_=0.0063). Sex-specific association analysis on the Flinders University replication cohort supported these findings, whereas results from the other two cohorts did not (Supplementary Fig. 7). Regardless, both rs3768617 and rs784257 showed significant sex heterogeneity of effect in meta-analyses combining discovery and replication samples (Supplementary Fig. 7). The discovery sample offered adequate power (∼0.8) to detect the SNP × sex interaction effects observed for these two markers with significant *P* values (Supplementary Table 14). Herein, we present the first evidence of genetic contribution to the sex-specific risk for FECD.

### Functional significance of major association results

To derive functional insights regarding the genes nearest the most significant SNPs from GWAS, we examined several biologic sources of information, including transcription factor, histone mark and expression quantitative trait loci (eQTLs) reference maps from public databases that did not include corneal tissue (HaploReg^26^; http://www.broadinstitute.org/mammals/haploreg/haploreg.php). Reasoning that similar regulatory features may be shared between the corneal endothelium and other cell types of neural crest origin, we placed greater emphasis if the evidence came from these cell types, but used gene expression profiles from normal corneal endothelium as a guide for interpretation. We then examined, by immunohistochemistry (IHC), patterns of relevant genes in corneal tissue sections from cases and controls using antibodies directed against specific gene products.

Examining transcriptomic profiles from normal corneal tissue and focussing on a 1-Mb interval centred on the best SNP for each locus, we note that *TCF4*, *ATP1B1* and *LAMC1* are highly expressed in corneal samples comprising only the corneal endothelium and DM (Supplementary Data 1). *LINC00970*, the lncRNA closest to *ATP1B1*, shows no expression, while *KANK4* shows minimal expression. *TCF4*, a basic helix–loop–helix transcription factor, was detected in the nucleus of residual endothelial cells of Fuchs case samples by IHC (Supplementary Fig. 8). No genotype data were available from the sample donors, and, therefore, we could not explore the influence of associated variants on gene expression in ocular tissue.

Our lead SNP in *KANK4*, rs79742895, was associated with promoter and enhancer mark enrichment in multiple tissues (Supplementary Data 2), conceivably altering motifs for transcription factors, hypermethylated in cancer1 and Y in Yang1. The cellular function of *KANK4* is poorly understood, but mutations in KANK proteins lead to steroid-resistant nephrotic syndrome^27^. Modelling of mutations via knockdowns illustrate that ∼50% of *kank2* morphant zebrafish develop periorbital oedema and kidney disease, which the authors ascribe to loss of kidney function. Knockdown analysis of the *KANK3* orthologue in *Caenorhabditis elegans* suggests a role in cell-to-cell contact and tissue integrity^28^. Distinct from TCF4, KANK4 immunostaining revealed that localization of KANK4 is mainly within the endothelial cytoplasm in both control and FECD samples (Supplementary Fig. 8). KANK family proteins may have a role in the regulation of actin stress fibres, which are hypothesized to hold the endothelial cell nuclei in place through cellular adhesion of the endothelial cytoplasmic layer^29^.

rs79742895, in *KANK4*, was also associated with an eQTL for the LINE-1 containing transposase domain containing 1 (*L1TD1*) gene 23.84 kb distal to *KANK4* (ref. 30). Similar to *KANK4*, *L1TD1* is only weakly expressed in the corneal endothelium (Supplementary Data 1). *L1TD1* is a RNA-binding protein involved in preserving pluripotency of stem cells via post-transcriptional regulation, showing high expression in pluripotent cells^31^. Functions of moieties that interact with *L1TD1* can be divided into three categories: RNA post-transcriptional modification, protein synthesis and gene expression. Emani *et al*.^31^ reported that *LAMC1* is involved in protein synthesis under control of *L1TD1*. No other variants with *r*^2^≥0.8 were located in linkage disequilibrium with rs79742895 and identified by HaploReg.

The strongest functional evidence for rs3768617 in *LAMC1* is with eQTLs. Eight entries with significant eQTLs were found to map within *LAMC1* or *LAMC2*, the vast majority in brain or nerve tissue (Supplementary Data 2). Thus, this variant, or others in linkage disequilibrium, appear to self-regulate the expression levels of *LAMC1* and *LAMC2*, both of which have a key role as cell adhesion proteins in basement membranes, such as DM^32^; laminins are asymmetrically expressed on the endothelial side of DM^33^. Knockouts of this gene cause failure of ureteric bud development, or reduction in kidney size, with an insufficiency of water transport postnatally^34^. An important feature of this model is the absence of basement membranes in the kidney early in development, and reduction in levels of integrin-α6 and *FGF2*, key elements of cellular growth. A similar *LAMC1*-knockout model in cardiomyocytes derived from human embryonic stem cells also exhibited lack of proper basement membrane formation, leading to inappropriate extracellular matrix deposition between cells that upset electrical signal conduction^35^. Analogous defects likely occur in the corneal endothelium and DM in FECD. Strengthening this hypothesis, IHC of control corneal endothelial samples pinpointed *KANK4* and *LAMC1* protein expression within the cytoplasm. In FECD cases, a decline in the number of endothelial cells resulted in concomitant decrease of *KANK4* and *LAMC1* positively stained cells (Supplementary Fig. 8), which retained cytoplasmic expression of the proteins.

rs1200114 maps to an intergenic region between *ATP1B1* and *LINC00970*, which does not show expression in the corneal endothelium. Conversely, *ATP1B1* shows high expression in the endothelium (Supplementary Data 1). This SNP shows no significant eQTLs in GTEx, but does show strong enrichment for enhancer and promoter marks, particularly in numerous brain tissues (Supplementary Data 2). The β-subunit of the multimeric Na^+^,K^+^-ATPase has a key function in structural integrity of the protein, and its functional maturity. It is a regulator of fluid balance, ion transport and maintains the cellular homeostatic equilibrium^36^. Lack of *ATP1B1* may lead to hypertonicity within the cornea and cause the corneal oedema seen in patients suffering from FECD.

Recognizing that *ATP1B1* represented our most significant GWAS signal after *TCF4*, we sought additional supporting evidence at the gene expression level. Unbiased mining of proteomic data by Poulsen *et al*.^37^ corroborated the expression of *LAMC1* and *ATP1B1* in disease-relevant tissues. By IHC using *LAMC1* and *ATP1B1* antibodies in three pairs of full-thickness corneas from FECD cases and controls, we confirmed expression of these proteins in the corneal endothelium (Supplementary Fig. 9).

## Discussion

We identified three novel loci, *KANK4*, *LAMC1* and *ATP1B1*, for FECD, and demonstrated cellular expression of *TCF4*. Our results support a multifactorial model for disease aetiology with strong predictive power (AUC=0.78 from an oligogenic risk-score model), although variants in *TCF4* alone do a commendable job of predicting risk in Caucasians. We confirm the strong association of variants such as rs613872 in *TCF4*, initially discovered by Baratz *et al*.^13^, and extend these results by demonstrating sex-specific effects. In contrast, we observed only modest association at loci previously identified through family-based and candidate-gene studies^10^,^11^,^12^,^38^,^39^,^40^.

Our data favour *LAMC1* as increasing risk in women, whereas *TCF4* increases risk in men. Alleles at both loci are quite common, and their precise role in mediating sex-specific risk remains to be elucidated. Taken together, these results suggest that these proteins have essential roles in the corneal endothelium; for instance, water transport, appropriate basement membrane maintenance, as well as tissue integrity and cell-to-cell contact. Disruption of these activities may lead to pathogenic processes characteristic of FECD. The primary function of the corneal endothelium is to maintain a state of relative dehydration, with hindrance of routine fluid transport leading to corneal oedema. We propose that all three genes regulate fluid transport, with *ATP1B1* having the most obvious role as it encodes a subunit of the sodium-potassium plasma membrane pump. *LAMC1* also has a role in normal basement membrane deposition, as shown in knockout models of the kidney and cardiomyocytes, possibly leading to thickening of the DM. Although functional data supporting an effect of rs3768617 on *LAMC1* expression exists in GTEx, this database does not include the cornea. Limited data on the function of *KANK4* means that its function can currently only be inferred by analogy to its paralogues, *KANK2* and *KANK3*, and is suggestive of maintenance of cell-to-cell contact and tissue integrity. In FECD, endothelial cells reduce in number and change shape to maintain regular contact with each other and to preserve the endothelial barrier to fluid entry into the corneal stroma^41^,^42^. Identification of additional genetic risk factors adds to our understanding of FECD, thus enabling development of novel therapies for this disabling disease.

## Methods

### Samples and phenotypes

Informed consent was obtained from all participants, in accordance with the Declaration of Helsinki, and all procedures were conducted after approval from the Institutional Review Boards at all participating centres.

FECD study subjects in the discovery sample were recruited through the FECD Genetics Multi-center Study, based at Case Western Reserve University, and at Duke University Eye Center (DUEC)^9^,^43^. The phenotype criteria are listed in Supplementary Table 1. The sample originally comprised 2,710 individuals, of whom 2,089 (1,412 FECD cases and 677 controls) were included in association analysis (Supplementary Table 2 and see below). In addition, we included 1,879 FECD control participants from the AREDS Refractive Error Substudy (dbGaP accession phs000001.v3.p1), originally collected for an age-related macular degeneration clinical trial, but subsequently included in a GWA study on refractive error; these subjects had no age-related macular degeneration, previous cataract surgery or evidence of corneal dystrophy^17^. Twenty-nine AREDS samples were submitted to both studies by both groups to ensure that genotype clustering was compatible between samples originally submitted by the AREDS group and those by the FECD Multi-center study. Supplementary Table 3 summarizes the characteristics of the discovery samples.

Severity of FECD was assessed in both discovery samples using a modified Krachmer grading scale^5^ (Supplementary Table 2). Discovery samples had undergone penetrating keratoplasty or Descemet stripping endothelial keratoplasty in at least one eye, or had at least grade 3 FECD by the modified Krachmer scale in at least one eye (although most individuals were symmetric). Because keratoplasty is an unreliable indicator of FECD severity (228 of 1,254 cases (18.2%) with no corneal oedema and a grade 4 or below)^44^, we conducted a sensitivity analysis restricting the case definition to include only FECD cases with histopathological verification of severe FECD (see below). Based on discussions between investigators at all three centres, the following inclusion criteria for controls have been established to determine subject eligibility from each ascertainment site: (1) 60 years of age or older, (2) normal cornea by slit-lamp biomicroscopy with no epithelial, stromal or endothelial abnormalities with the exceptions of (a) arcus senilis and Vogt's limbal girdle; (b) cornea scar from infection or penetrating trauma; (c) pterygium; inactive superficial vascularization of the epithelium or subepithelial zone; (3) previous cataract, glaucoma or retina–vitreous surgery was allowed, as long as the preoperative record indicated no evidence by slit-lamp examination of guttae or FECD and postoperative exam also showed no evidence; and (4) previous intraocular laser surgery (such as iridotomy, trabeculoplasty or pan retinal photocoagulation) was allowed with same caveats as intraocular surgery.

A total of 1,449 samples (671 FECD cases and 778 controls) from three study centres were included in the replication phase (Supplementary Table 5). Three centres participated: the University of Iowa (Iowa City, IA, USA), Flinders University (Adelaide, SA, Australia) and Johns Hopkins (JHU) (Baltimore, MD, USA). All three centres required controls to be 50 years or older, and cases to have Krachmer grade 1 (ref. 5) (modified Krachmer grade 2) FECD or higher (Supplementary Table 2). We used the grading scheme provided by the replication cohorts and converted it to grades equivalent to the scale used for the discovery cohort. Detailed below are the grading criteria used by each cohort. Because of the scarcity of available replication samples, the case definition is in general less strict than for the discovery sample.

The University of Iowa cohort comprised 113 patients with FECD and 113 control subjects, all of European ancestry. All participants underwent detailed ophthalmic evaluation that included slit-lamp biomicroscopy. Patients were considered to have FECD if they had more than 12 central guttae or if they had histopathologic confirmation following corneal transplantation (keratoplasty) for corneal oedema^45^. Control subjects were examined by a board-certified ophthalmologist and did not have signs of FECD. A sample of ∼10 ml blood was collected from each study participant.

The Flinders University cohort comprised 190 patients with FECD. Additionally, genomic DNA from 282 unrelated, unaffected South Australian residents aged over 50 years, recruited previously for use as controls in ocular genetic studies in our laboratory, was available for this study^46^,^47^,^48^.

The cases were Caucasian Australian patients diagnosed with modified Krachmer scale grade 2 or above FECD who were recruited by ophthalmologists at the Flinders Eye Clinic (Adelaide, SA, Australia) and the Royal Victorian Eye and Ear Hospital (Melbourne, Victoria, Australia). Venous blood samples were collected from 190 patients and genomic DNA extracted using QIAamp DNA Blood Maxi Kit (Qiagen, Doncaster, Victoria, Australia) according to the manufacturer's protocol.

This study was approved by the Human Research Ethics Committees of the Flinders Medical Centre/Flinders University of South Australia (Adelaide, SA, Australia), and the Royal Victorian Eye and Ear Hospital. The research was conducted in accordance with the National Health and Medical Research Council, Australia guidelines.

The JHU cohort consisted of 368 Caucasian FECD cases and 380 ethnically matched control subjects. All participants underwent a detailed ophthalmic evaluation that included slit-lamp biomicroscopy. Affectation status and disease severity were determined with the Krachmer scale^5^. Positive disease status was indicated if the patient had a minimum modified Fuchs Krachmer grading score of 2 in at least one eye. The inclusion criteria for control subjects consisted of a minimum age of 58 years and no signs or symptoms of FECD by slit-lamp biomicroscopy. A 10-ml blood sample was collected from each study participant. DNA was extracted using the Gentra Puregene Blood Kit (Qiagen, Santa Clara, CA, USA).

### Genotyping

Genotyping for the discovery samples was performed on the Illumina HumanOmni2.5-4v1_H array at the Center for Inherited Disease Research (CIDR, Baltimore, MD, USA). DNA samples on 96-well plates were balanced by FECD status, collection site (Case Western Reserve University multi-center versus DUEC) and sex.

A total of 2,841 FECD case–control samples, including duplicates, were submitted for genotyping, of which 2,818 passed initial genotyping QC by CIDR. The 1,922 AREDS control DNA samples, including 29 cross-study duplicate pairs and 10 internal duplicate pairs, were processed separately at CIDR, but genotype calling was performed with both sets together.

Eighteen SNPs were chosen for follow-up based on the genetic structure and annotation information for the most significant results from the Omni2.5M and imputed GWAS within each region yielding *P*<5 × 10^−8^ for association for at least one marker. The LD structure was characterized, using genotype data from our FECD controls only, among the five SNPs with the most significant associations from the Omni2.5M data, and at least five SNPs from the imputed data. From each region we chose two or more SNPs for replication: the most significant SNP plus one or more additional SNPs and, where available, highly significant exonic variants. Follow-up SNPs were chosen to minimize LD within association peaks; SNPs within coding regions were chosen preferentially. Samples were genotyped for 18 SNPs in or near *KANK4* (rs12082238, rs12058486, rs79742895), *ATP1B1* (rs1200114, rs1200115), *LAMC1* (rs2296292, rs3768617, rs1413386, rs20560, rs20561), *CFH* (rs2274700, rs1329428), *SLC25A22* (rs12223324, in *PDDC1*; rs4963153; rs1138714, in *PNPLA2*) and *TCF4* (rs72932713, rs11659764, rs784257). The well-characterized *TCF4* SNP rs613872 was not specifically selected for follow-up, but genotype data were available for the SNP in the Flinders University sample. rs2296292 and rs20560 in *LAMC1* were chosen specifically because they were coding variants (A592A and R1376R, respectively).

For the University of Iowa replication sample, DNA was extracted using the Gentra Puregene Blood Kit (Qiagen, USA). Samples were submitted to LGC Genomics (Petaluma, CA, USA) for genotyping using the KASPar assay, an end-point polymerase chain reaction (PCR) assay using two competitive, allele-specific forward primers and a common reverse primer. The average call rate over 17 assays was 98.5%; rs2274700 failed to genotype.

For the Flinders University sample, genotyping data for the replication SNPs were obtained for 190 FECD cases and 282 unaffected controls. All the SNPs were genotyped in cases and controls using iPLEX GOLD chemistry (Sequenom, Herston, QLD, Australia) on a MassARRAY Compact Spectrometer (Sequenom).

For genotyping of the Johns Hopkins University sample, the PCR was performed in 5-μl volumes containing 10 ng genomic DNA, 2.5 μl SNP genotyping master mix (TaqMan; Applied Biosystems, Foster City, CA, USA) and 0.125 μl genotyping assay mix (TaqMan; Applied Biosystems). Reactions for all nine SNPs were amplified independently in a thermocycler (9700; Applied Biosystems). The cycling parameters consisted of 2-min incubation at 50 °C and denaturation at 95 °C for 10 min, followed by 40 cycles of 10 s at 95 °C and 1-min elongation at 72 °C with a final 10-min extension at 72 °C. Amplified products were analysed for the enrichment of specific alleles (ABI 7900HT Sequence Detection System; Applied Biosystems).

We assayed the CTG18.1 repeat in intron 2 of *TCF4* (refs 16, 21, 49) on a subsample of 664 individuals from the discovery cohort by classifying repeats as ‘unexpanded' (≤40 copies of the repeat) and ‘expanded' (>40 copies)^49^. An initial analysis was performed using a short tandem repeat assay^21^ by amplifying the triucleotide repeat with primers 5′-FAM-AATCCAAACCGCCTTCCAAGT-3′ and 5′-AATCCAAACCGCCTTCCAAGT-3′ followed by sizing of PCR products by capillary electrophoresis on an Applied Biosystems 3730 Genomic Analyser with LIZ500 size standards^21^. Samples not showing evidence of two distinct alleles were further analysed using a repeat-primed PCR assay^21^ incorporating an internal primer 5′-TACGCATCCCAGTTTGAGACGCAGCAGCAGCAGCAG-3′ and downstream primer 5′-TACGCATCCCAGTTTGAGACG-3′ (ref. 21) to check for an expanded allele not detected by the short tandem repeat assay.

### GWAS quality control

Genotype data were subject to intensive quality control by the Quality Assurance/Quality Control analysis team at the University of Washington Genetics Coordinating Center. Genotype concordance between duplicate samples was high; the median discordance rate over 102 duplicate pairs was 1.5 × 10^−4^. All samples passing quality control had call rates above 98%, and the median call rate was 99.75%.

Relatedness between pairs of samples was estimated by means of the method-of-moments approach^50^ implemented in the R package SNPRelate, on a subset of 124,583 autosomal SNPs in low linkage disequilibrium. A total of 15 samples were omitted on account of unresolved identity issues, including disagreement of annotated and genetic sex and of unexpected and unresolvable relationships. Samples were classified as ‘related' if their estimated kinship coefficient was >1/32 (equivalent to half the expected value for first cousins). Association analysis included a total of 2,089 unique, unrelated samples of European descent from the FECD Multi-center and DUEC samples that met the consortium criteria for FECD case or control status.

Genetic sex was determined by means of X-chromosome heterozygosity and the mean fluorescence intensity of the X and Y chromosomes.

Chromosomal anomalies, including subchromosomal aneuploidy and mosaic uniparental disomy, were detected using circular binary segmentation on B allele frequencies or runs of homozygosity^51^. Anomalies >5 megabasepairs (Mb) in length and entire chromosomes carrying >10 Mb of anomalies were annotated. Genotypes within these regions from the individuals carrying the anomalies were omitted from association analyses. Anomalies of the X chromosome were annotated but not filtered.

Ancestry was estimated by means of principal components analysis (PCA) by the approach of Patterson *et al*.^52^ as implemented in the R SNPRelate package. PCA was initially performed on founder individuals using a subset of 133,530 autosomal SNPs pruned for linkage disequilibrium. Principal components were determined for family members by the method of Zhu *et al*.^53^ Nine individuals of putative European descent with outlying values for the first two principal components were omitted from association analysis.

Genotypes were initially obtained on 2,443,177 SNPs, of which 2,416,694 passed CIDR technical filters. SNPs were excluded under the following quality control filters: minor allele frequency=0 in the sample; cluster separation<0.2; missing rate≥2%; more than one discordant call among 158 study duplicates, including 27 HapMap sample pairs; any discordant calls within the FECD Multi-center^9^ and AREDS^17^ cross-study duplicates; more than one Mendelian incompatibility; a *P* value of <10^−4^ from an exact test of Hardy–Weinberg proportions; sex difference in allele frequency≥0.2; and sex difference in heterozygosity>0.3. A total of 2,111,249 SNPs passed all quality filters, including 1,449,159 with minor allele frequency≥0.02.

### Imputation

Imputation of untyped variants in the 1000 Genomes Phase 1 reference panel, version 3, on all study samples passing quality control filters was carried out at the University of Washington Genetics Coordinating Center. Prephasing of 1,587,835 autosomal and non-pseudoautosomal, X-linked SNPs overlapping with the reference panel was conducted by means of SHAPEIT2 (ref. 54) with 200 conditioning states. Imputation was performed over 5-Mb segments with a 500-kb buffer using IMPUTE2 (ref. 55), based on a cosmopolitan reference panel of all 1,092 samples from the Phase 1 integrated variant set. Imputed genotypes within chromosomal anomalies (see Sample Quality Control, above) were marked as missing in the final output. Imputation was restricted to SNPs with at least two copies of the minor allele within the pooled European continental group; all indels and structural variants were excluded. Imputation yielded 12,660,865 SNPs, including 354,144 X-linked SNPs.

### Statistical analysis

Association between FECD status and 1,449,159 genetic markers from the Omni2.5 panel was tested using logistic regression as implemented in the R GWAStools package, including age, sex and the first six principal components from the PCA as covariates, using an additive inheritance model in the number of alleles coded ‘A' by Illumina (not necessarily the minor allele). Significance of association was measured using a Wald test. In testing X-linked SNPs, male genotypes were coded as 0 and 2 (for *BY* and *AY*, respectively). The overall genomic control parameter *λ* (ref. 56) was 1.018. Because *λ*-values below 1.05 are generally considered not significantly >1, no adjustment for genomic control was performed.

Association testing was also performed on 8,680,745 imputed SNPs with IMPUTE2 information quality metrics>0.5 using the same statistical model. Here, the genetic predictor was the dosage of the minor allele. The values of *λ* over all SNPs and omitting the *TCF4* region were 1.033 and 1.028, respectively (Supplementary Fig. 2).

We conducted subset analyses by (1) stratifying the sample by sex, and (2) restricting the FECD cases to those confirmed by histopathological examination (*n*=787), comparing to all controls (Supplementary Table 3). Association tests were run by the same logistic regression model as described above, except that sex was not included as a covariate in the sex-stratified analyses. Both genotyped and imputed markers were analysed. Evidence for heterogeneity between men and women was assessed by a *χ*^2^ goodness-of-fit test comparing normalized sex-specific effect estimates with the pooled estimate^57^.

Genotypes from the replication samples were analysed by sample using PLINK^50^ or R. *χ*^2^ and Fisher's exact tests were conducted to assess allelic and genotypic associations. Allele frequencies in FECD cases and controls were compared using Fisher's exact test. Meta-analysis over three replication samples (Supplementary Table 6) was performed by the inverse-variance weighting method, from log ORs and 95% CIs estimated by the OR function in the R package EpiTools. Because genome-wide marker data were unavailable for the replication samples, we did not adjust for population structure by means of PCs.

A genetic risk metric was constructed from genotypes of the discovery sample only at the most significantly associated SNP at each of the six candidate gene regions (Fig. 1), coded so that the effect allele was the risk-conferring allele, summing the product of the number of risk alleles at each locus with the log of the OR for the discovery cohort as listed in Table 1. The area under the curve (AUC) and its 95% confidence interval was calculated, and AUCs of different receiver operating characteristic (ROC) curves were compared, using the R package pROC. Sex-specific genetic risk scores were calculated by the same procedure, using effect estimates derived from Supplementary Table 13.

Pairwise SNP:SNP interactions were tested among pairs of SNPs from Fig. 1 by including two genotypes and an interaction term in the association model for the discovery sample.

### Gene expression and IHC

Surgical specimens of corneal endothelium and Descemet's membrane (CE+DM) complex were obtained from patients undergoing keratoplasty because of advanced FECD and placed directly into *RNAlater* Solution (Life Technologies, Mulgrave, VIC, Australia). The RNA from these tissues was used for sequencing. Equivalent control specimens were obtained through the Eye Bank of South Australia (Flinders Medical Centre, Adelaide, SA, Australia). The specimens were placed in RNA*later* Solution immediately after surgery or dissection, and stored at 4 °C for at least two days. Then RNA*later* solution was removed and the specimen stored at −80 °C for later gene expression analysis.

Normal, eye-bank-derived, post-mortem corneas in Optisol GS from three unaffected donors were obtained for gene expression analysis from Eversight Ohio (Cleveland, OH, USA). The storage solution was discarded and tissues were placed into *RNAlater* Solution and frozen on receipt. For each of the six loci from the initial GWAS, the set of GENCODE genes (accessed through Ensembl Biomart, http://www.ensembl.org) within and overlapping an interval of 1 Mb centred on the most significant marker was included in the analysis. RNA was isolated using the RNeasy Plus Kit (Qiagen, USA) according to the manufacturer's protocol. RNA was quantified using Qubit Picogreen fluorescent assay (Life Technologies, Carlsbad, CA, USA) and RNA integrity was checked with the Bio-Rad Experian/Agilent Bio-analyser. Samples passing initial quality control were subject to rRNA depletion with RiboZero Gold (Epicentre, San Diego, CA, USA) as per the manufacturer's instructions. rRNA-depleted fractions were used as input for library preparation using ScriptSeq Gold Low Input Library Preparation Kit (Epicentre). All libraries were quantified using the Qubit Picogreen fluorescent Assay (Life Technologies) and analysed with the Bio-Rad Experion system. The libraries were further quantified with quantitative PCR using the KAPA Library Quantification Kit (KapaBiosystems, Wilmington, MA, USA). Sequencing was performed for 100 bp paired-end on the Illumina HiSeq 2500. The reads were mapped to the reference human genome (hg38) using tophat (https://ccb.jhu.edu/software/tophat/index.shtml). Read counts were performed for features in the GenCode version 21 database using htseq-count (http://www-huber.embl.de/users/anders/HTSeq/doc/count.html). The expression level was normalized for each gene feature by calculating reads per kilobase per million reads. We defined ‘high expression' as >10 reads per kilobase per million reads averaged over the three replicates.

For the primary immunohistochemical analysis, formalin-fixed, paraffin-embedded sections, 4–5 μm thick, of five FECD-affected and five normal corneas were used. Immunohistochemical labelling on sections of affected and normal corneas was performed as follows^20^,^58^. Primary antibodies were applied to each section and incubated for 1.5 h in a chamber at 37 °C. The dilutions were as follows: ATP1B1 (LifeSpan BioSciences; LS-B235), 1:1,000; LAMC1 (LifeSpan BioSciences; LS-B7354), 1:50; TCF4 (LifeSpan BioSciences; LS-B1570), 1:200; KANK4 (Abcam; ab121410), 1:100. We used goat anti-rabbit horseradish peroxidase (Abcam; ab6721) diluted 1:400 and goat anti-mouse horseradish peroxidase (Fisher Scientific; PI31430) diluted 1:200 as the secondary antibodies. Sections were processed with DAB Quanto (Thermo Fisher Scientific; TA-060-QHDX), were counterstained with haematoxylin and were mounted in Cytoseal XYL (Thermo Scientific, 8312-4). Images were captured on an Olympus BH-2 microscope (Olympus, Tokyo, Japan) fitted with a Canon EOS6D 20.2 Megapixel Digital Camera (Canon, Tokyo, Japan) using DSLR Remote Pro Imaging Software (Breeze Systems, Surrey, UK).

Labelling on sections of affected and normal corneas for confirmatory immunohistochemical analysis was performed as above^20^, but without any antigen retrieval. Sections were hybridized with the mouse anti-human ATP1B1 (clone M17-P5-F11, catalogue no. PIEMA3-930, Thermo Scientific Pierce, supplied by SABiosciences; 1:1,000 dilution) or rat anti-human LAMC1 (catalogue no. AB80580, Sapphire Bioscience, Waterloo, NSW, Australia; 1:50 dilution) monoclonal antibody (1:50) at 4 °C overnight. This was followed by hybridization with the NovoLink Polymer complex reagent (Leica Microsystems, Bannockburn, IL, USA). Primary antibody binding was detected with Liquid DAB+ substrate Chromogen System (K3468; Dako Australia). Sections were counterstained with haematoxylin and mounted in DePeX (Merck KGaA, Darmstadt, Germany). Labelling was imaged on an Olympus BX50 microscope fitted with QImaging Micropublisher RTV 5 Megapixel Digital Camera using QCapture Imaging Software (Olympus)^20^.

### Web resources

Software:

GWASTools: http://www.bioconductor.org/packages/devel/bioc/html/GWASTools.html

SNPRelate: http://cran.r-project.org/web/packages/SNPRelate/index.html

PLINK: http://pngu.mgh.harvard.edu/~purcell/plink/index.shtml

IMPUTE2: https://mathgen.stats.ox.ac.uk/impute/impute_v2.html

SHAPEIT: https://mathgen.stats.ox.ac.uk/genetics_software/shapeit/shapeit.html

EpiTools: http://medepi.com/epitools

### Data availability

We have deposited all genotype data supporting our findings from the discovery cohort in the Database of Genotypes and Phenotypes (dbGaP), with accession code phs000421.v1.p1. Other data that support our findings are available from the authors by request; see author contributions and their published references for specific data sets.

## Additional information

**How to cite this article:** Afshari, N. A. *et al*. Genome-wide association study identifies three novel loci in Fuchs endothelial corneal dystrophy. *Nat. Commun.* **8**, 14898 doi: 10.1038/ncomms14898 (2017).

**Publisher's note:** Springer Nature remains neutral with regard to jurisdictional claims in published maps and institutional affiliations.

## Supplementary Material

Supplementary Information Supplementary Figures, Supplementary Tables and Supplementary References

Supplementary Data 1 Gene expression over a 1-megabase window near six FECD-associated loci in normal corneal tissue.

Supplementary Data 2 Promoter, Enhancer and eQTL Annotations for Major Loci

## Figures and Tables

**Table 1:** Major results from the FECD genome-wide association study.

**Gene**	**SNP**	**Chr**	**Pos**	**All.**	**Discovery cohort (*****n*****=3,968)**				**Replication Cohorts (*****n*****=1,449)**		**Combined (*****n*****=5,417)**		
					**RAF case**	**RAF ctrl**	**OR (95% CI)**	***P***_**disc**_	**OR (95% CI)**	***P***_**repl**_	**OR (95% CI)**	***P***_**combined**_	**Q*****p***
*KANK4*	rs79742895	1	62,782,860	C/T	0.0889	0.0474	2.07 (1.69, 2.54)	2.5 × 10^−12^	2.18 (1.36, 3.50)	1.2 × 10^−03^	2.09 (1.73, 2.52)	1.2 × 10^−14^	0.83
*ATP1B1*	rs1200114	1	169,060,489	G/A	0.4277	0.3411	1.43 (1.29, 1.57)	8.0 × 10^−13^	1.53 (1.30, 1.79)	1.7 × 10^−07^	1.45 (1.34, 1.58)	9.9 × 10^−19^	0.48
*LAMC1*	rs3768617	1	183,092,500	A/G	0.3553	0.4214	0.75 (0.68, 0.82)	8.8 × 10^−09^	0.62 (0.53, 0.72)	2.1 × 10^−09^	0.71 (0.65, 0.77)	6.9 × 10^−16^	0.26
*CFH*	rs2274700	1	196,682,947	T/C	0.4287	0.3709	1.31 (1.19, 1.44)	3.4 × 10^−08^	1.08 (0.91, 1.27)	3.8 × 10^−01^	1.25 (1.15, 1.36)	1.8 × 10^−07^	0.097
*SLC25A22*	rs12223324	11	772,701	A/G	0.4826	0.5445	0.77 (0.70, 0.85)	2.1 × 10^−07^	0.92 (0.79, 1.07)	2.7 × 10^−01^	0.81 (0.75, 0.88)	7.3 × 10^−07^	0.24
*TCF4*	rs784257	18	53,397,199	A/G	0.4779	0.1796	5.77 (5.05, 6.60)	3.1 × 10^−146^	3.89 (3.30, 4.59)	6.3 × 10^−59^	4.94 (4.45, 5.48)	2.5 × 10^−200^	0.0041

All., alleles with reference allele first; CI, confidence interval; Chr, chromosome; OR, odds ratio; Q*p*, *P* value from Cochran's *Q* test for heterogeneity; Pos, hg37 physical map position; RAF case and RAF ctrl, reference allele frequency in cases and controls, respectively.
