# Supplementary material for: Genome-wide association study identifies three novel loci in Fuchs endothelial corneal dystrophy
Source: Nat Commun. 2017 Mar 30;8:14898. doi: 10.1038/ncomms14898 (PMC5379100; doi:10.1038/ncomms14898)
Supplement: Supplementary Information — Supplementary Figures, Supplementary Tables and Supplementary References [file ncomms14898-s1.pdf]

Supplementary Figure 1. Locus-specific plots of regions showing genomewide significant association in the FECD GWAS.

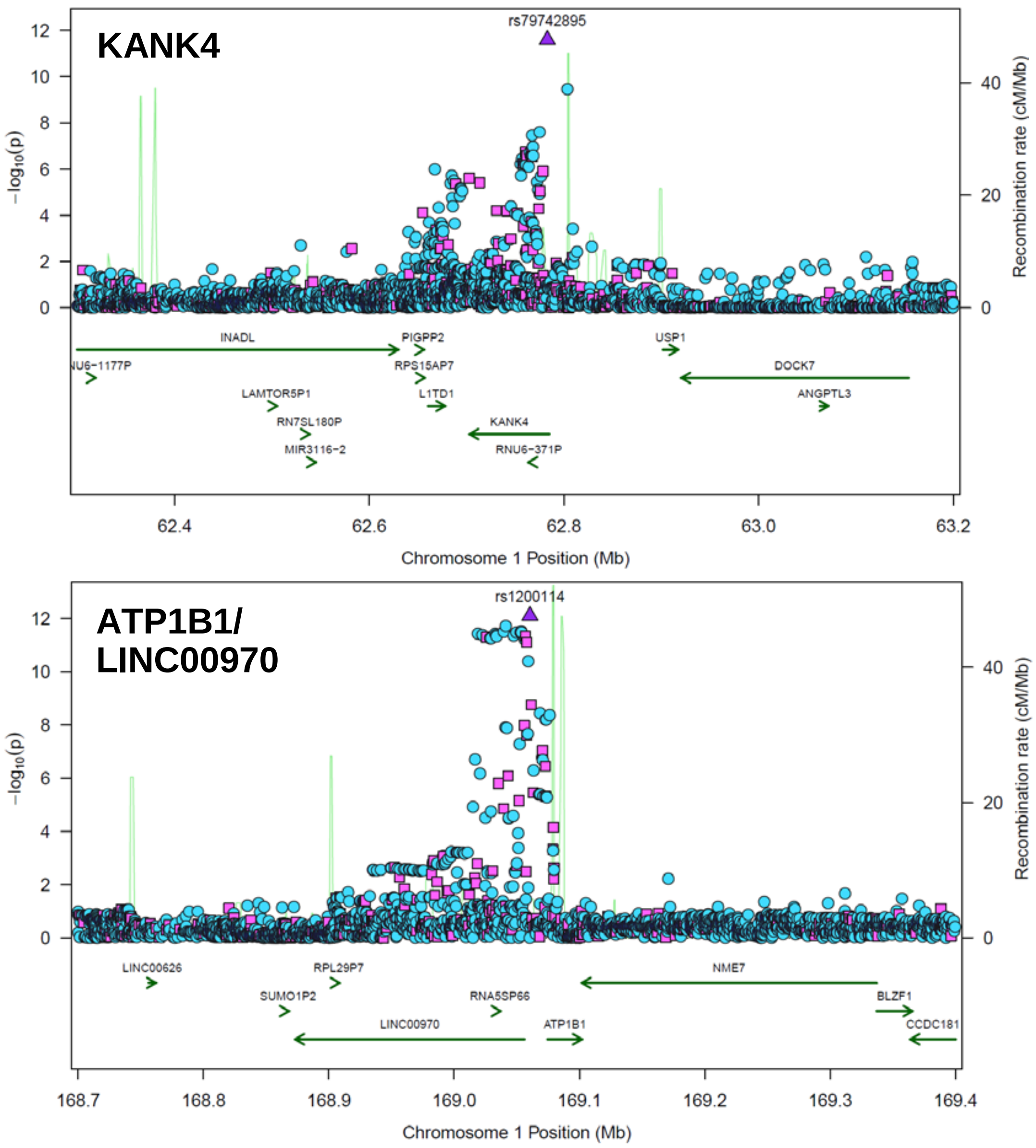

Supplementary Figure 1, cont.

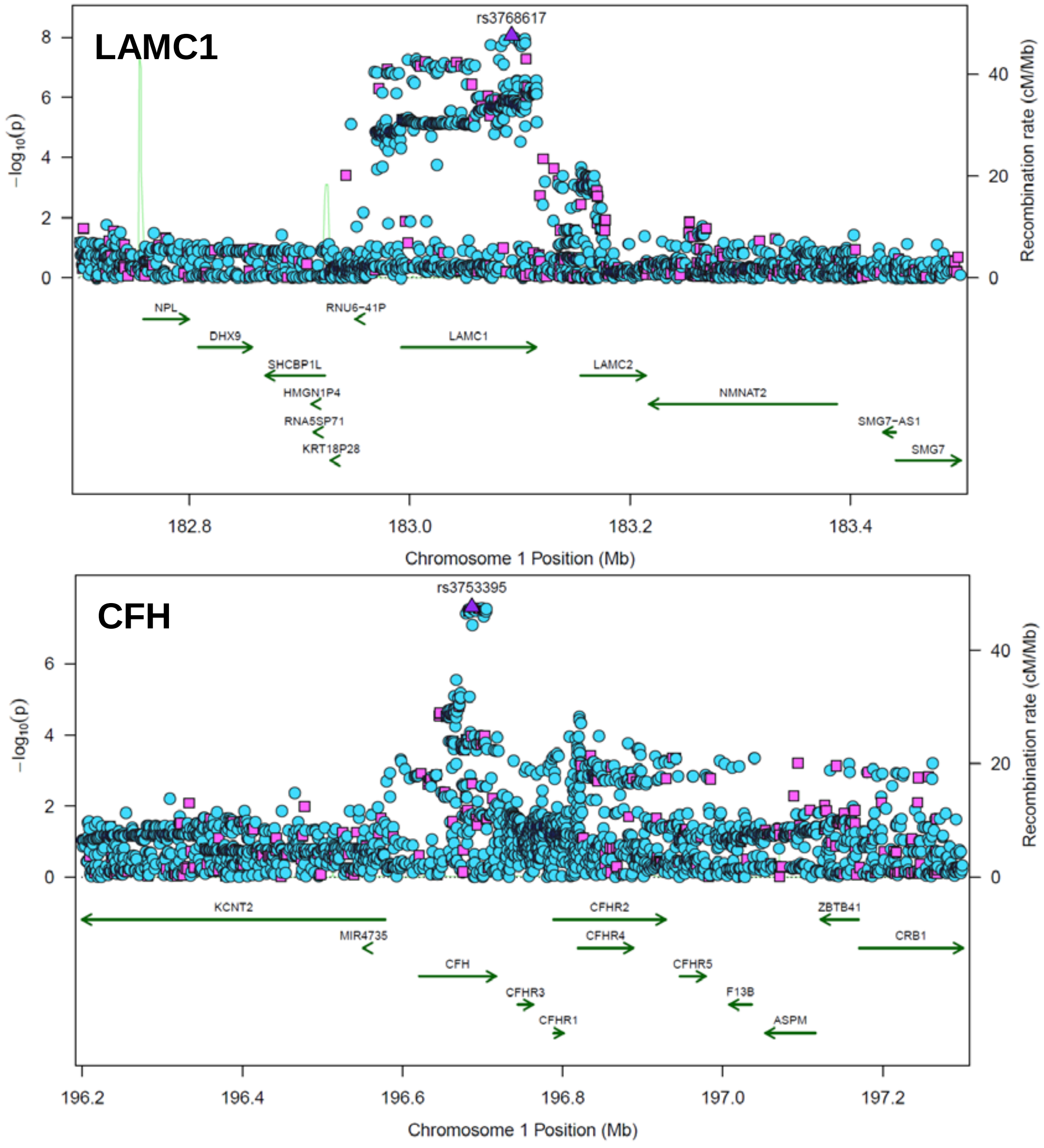

Supplementary Figure 1, cont.

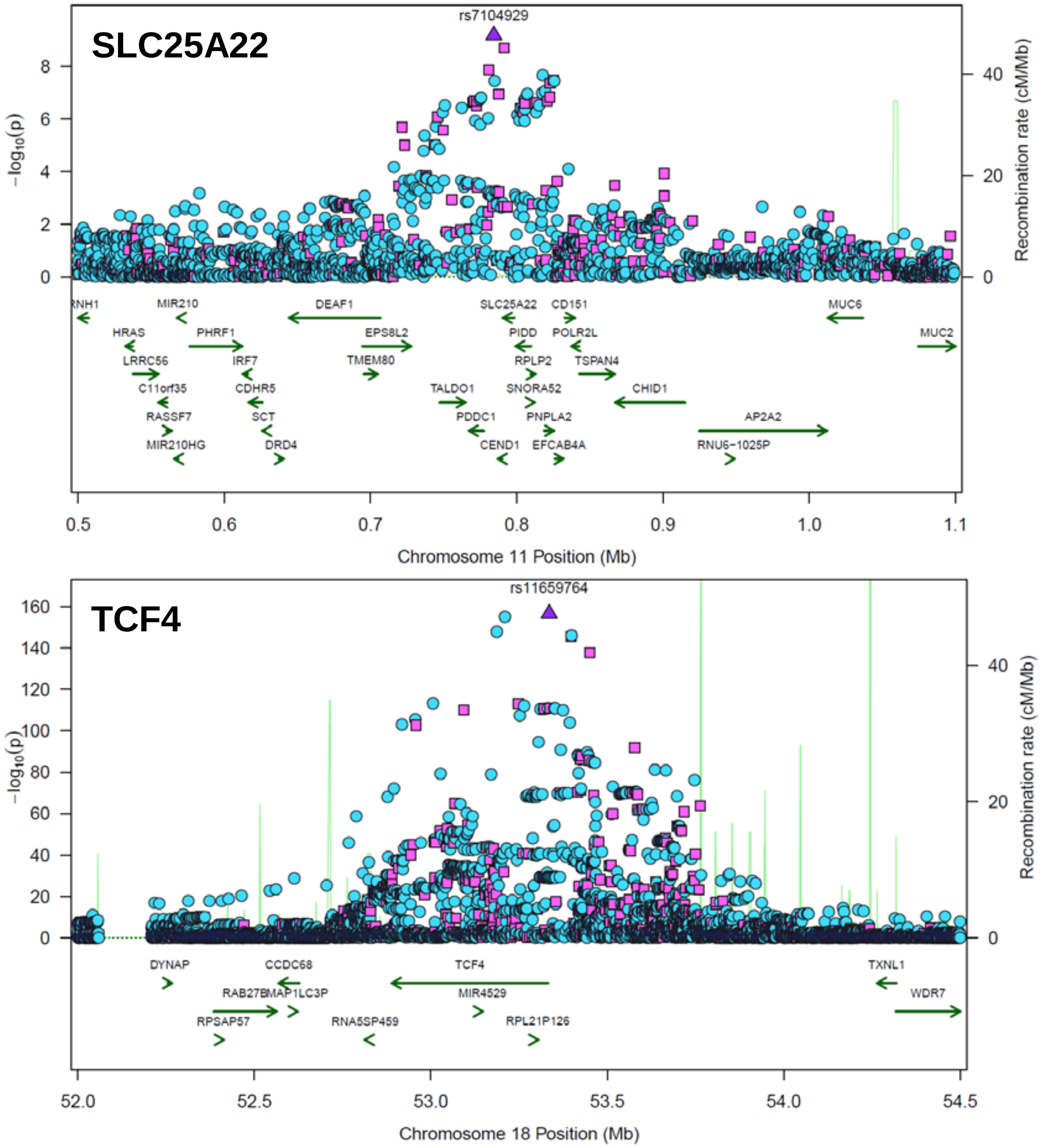

Genotyped (magenta squares) and imputed (cyan circles) markers are shown, and the index marker is indicated by a purple triangle; recombination rate is depicted in light green, genes (dark green arrows) are shown with direction of transcription.

Supplementary Figure 2. Quantile-quantile plots of association *p* values from the imputed GWAS for FECD.

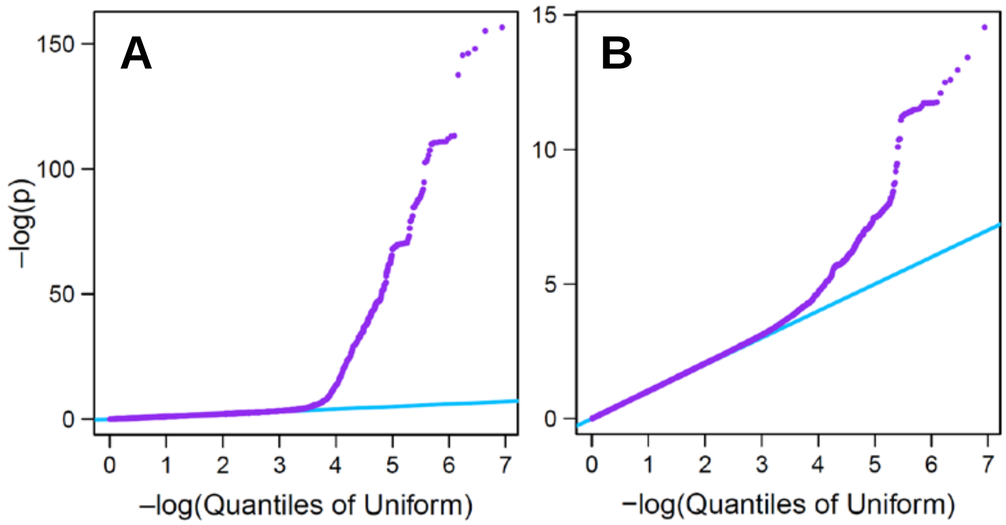

A, including all markers; **B,** excluding the *TCF4* signal. The genomic control parameter (λ) is 1.033 and 1.028 for (**A**) and (**B**), respectively.

Supplementary Figure 3. Forest plots for the major validated SNPs.

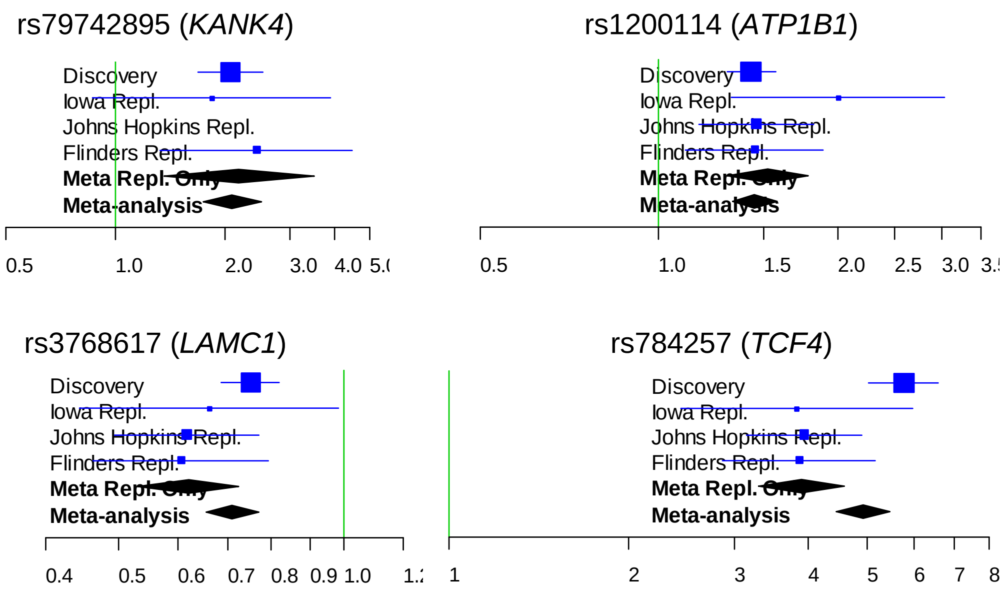

Estimated odds ratios (ORs) and 95% confidence intervals (CIs) from individual cohorts are indicated by blue boxes and horizontal lines, respectively. The area of the boxes is proportional to sample size. Results from meta-analyses are shown as black diamonds centered on the OR with width equal to the 95% CI. Meta Repl. Only, meta-analysis over the replication cohorts only; Meta-analysis, meta-analysis over discovery and replication cohorts.

Supplementary Fig. 4. Plot of log odds ratios from GWAS including only histopathology-verified cases vs. GWAS with all cases.

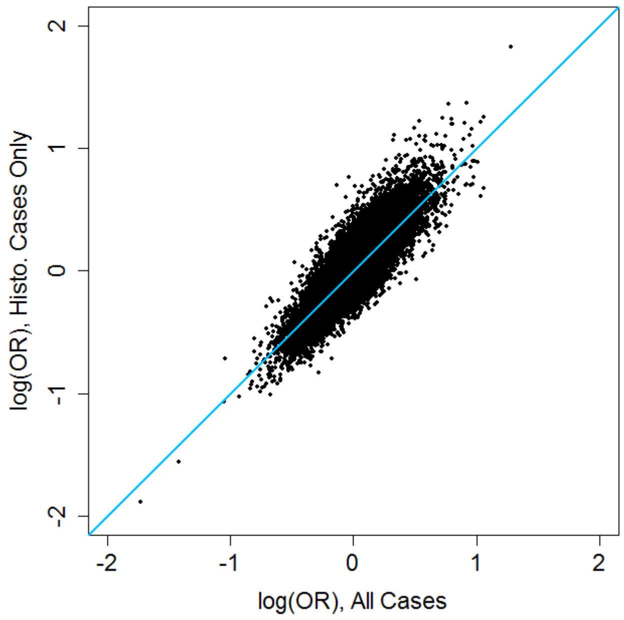

Overall, effect sizes are of greater magnitude in the results including only histopathology-verified cases (estimate of slope = 1.00839, SE = 0.00021; line of equality is drawn in blue).

Supplementary Fig. 5. Quantile-quantile plots of sex-specific GWAS on the discovery cohort.

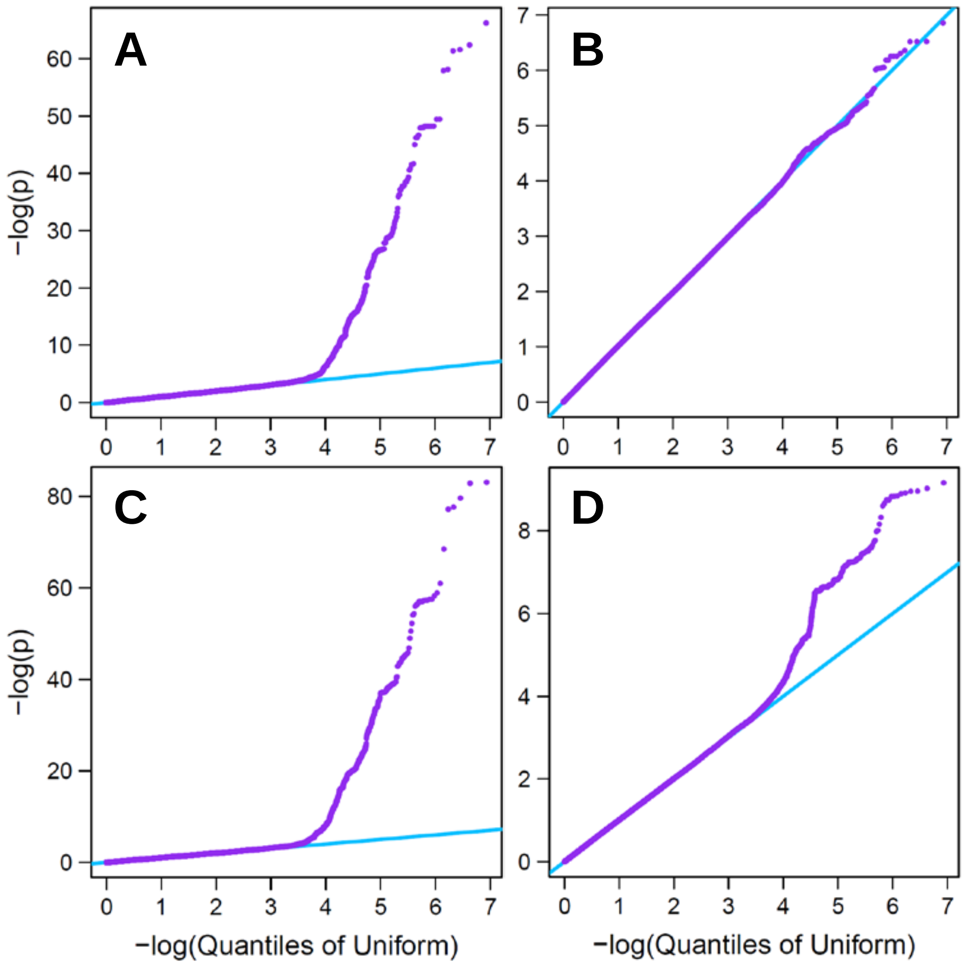

The genomic control parameter (λ) is 1.021 and 1.026 for men-only (**A,** all markers; **B,** without *TCF4* region) and women-only (**C,** all markers; **D,** without *TCF4*) analyses, respectively.

Supplementary Fig. 6. Manhattan plots of sex-specific GWAS on the discovery cohort.

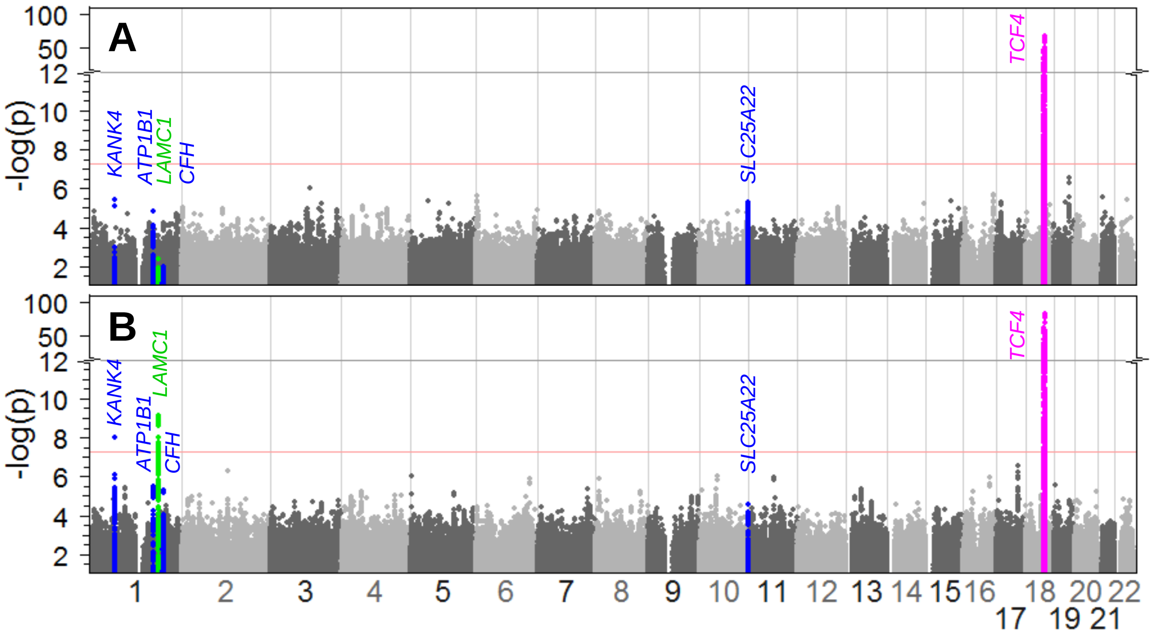

A, men only; **B,** women only. Colored points are within one of the six genomewide significant loci from the sex-combined analysis (**Fig. 1, Table 1**), as indicated.

Supplementary Figure 7. Forest plots for sex-specific meta-analysis of the validated association peaks.

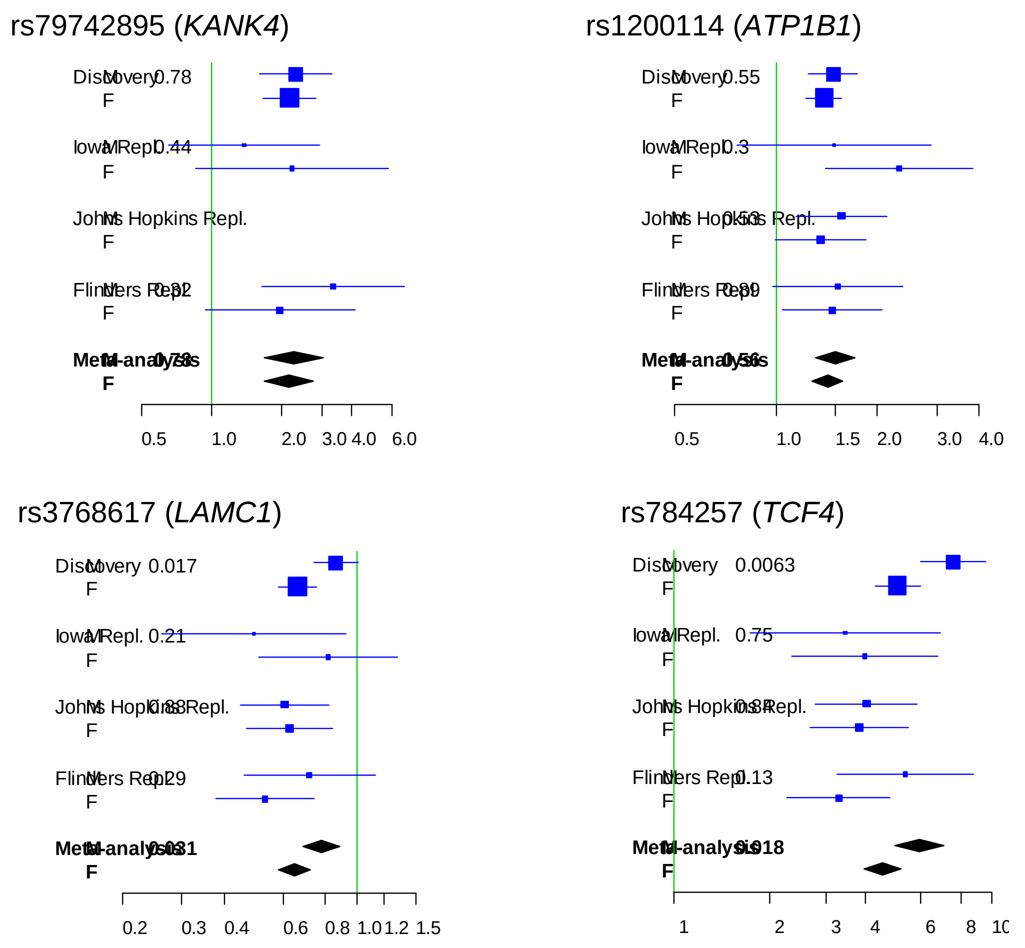

Results from mean-only (M) and women-only (F) association analyses are shown for each cohort and meta-analysis. See **Supplementary Figure 3** for explanation of symbols.

Supplementary Figure 8. Primary immunohistochemical labeling for the TCF4, KANK4, LAMC1 and ATP1B1 proteins.

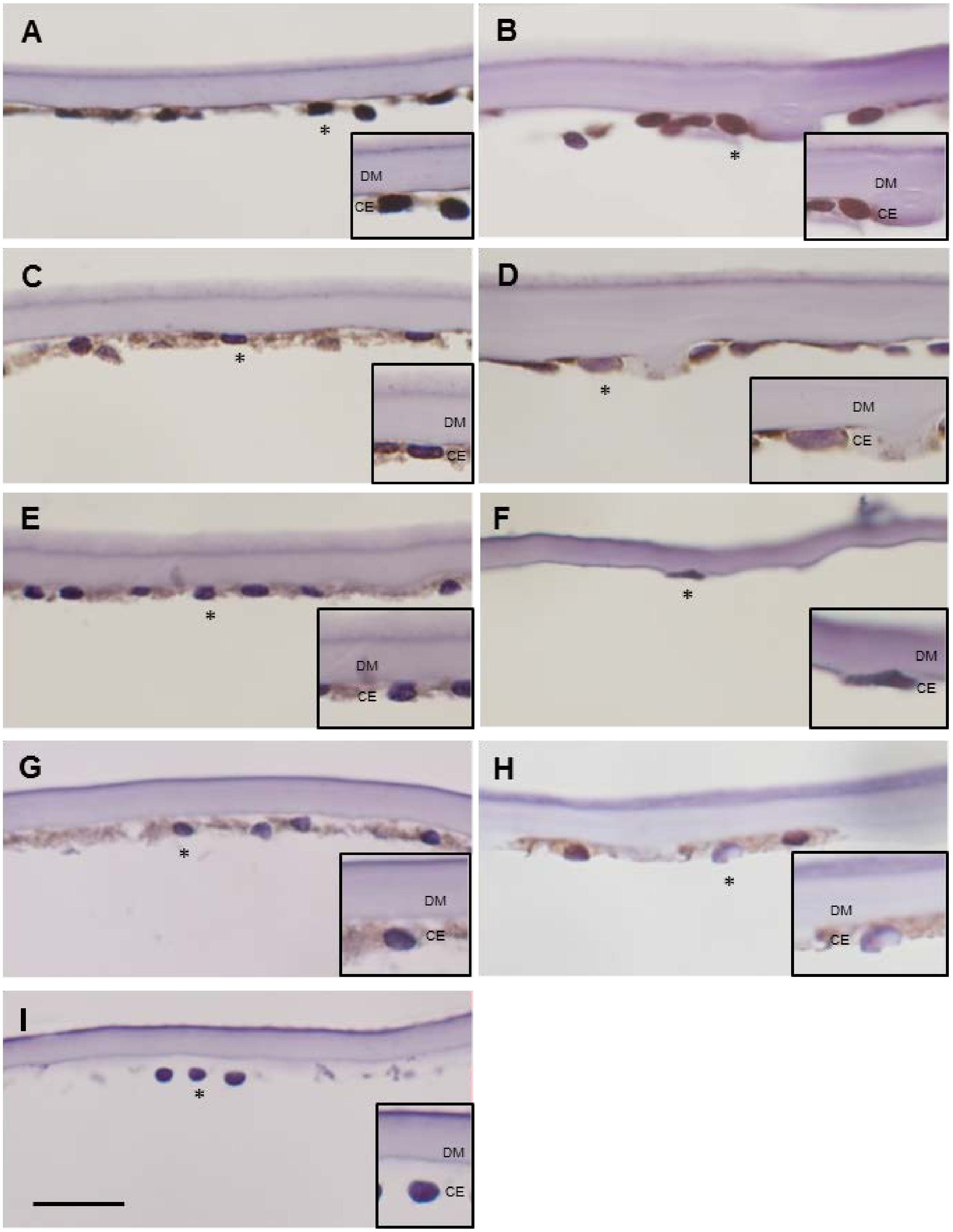

Corneal sections (DM + endothelium) are shown for FECD patients (left panels) and normal controls (right panels). The sections were immunostained with rat anti-human primary antibodies and goat anti-rat IgG secondary antibodies (see Materials and Methods). More intense TCF4-positive labeling (**A, B**) was observed in endothelial cell nuclei than the cytoplasm. KANK4 staining (**C, D**) showed the opposite, with more staining noted in the cytoplasm. LAMC1 (**E, F**) and ATP1B1 (**G, H**) positive labeling concentrated in the cytoplasm in the endothelial cells. The negative control (no primary antibody) is shown in panel **I**. For each antibody, positive labeling was observed in the corneal endothelium (CE) in both diseased and normal corneas. However, less frequent labeling was both expected and observed in the diseased cornea because of partial loss of the endothelial cells. Absence of similar labeling in the negative control section indicates that labeling in the presence of the anti-human primary antibodies utilized in this study were specific. Representative images from experiments in five independent pairs of corneas from FECD patients and controls are presented. The scale bar (panel **I**) represents 30 µm. Images are at 100× magnification; insets at 200×. An asterisk in each panel indicates a nucleus also present in the inset. DM, Descemet's membrane; CE, corneal endothelium.

Supplementary Figure 9. Validation of immunohistochemistry results.

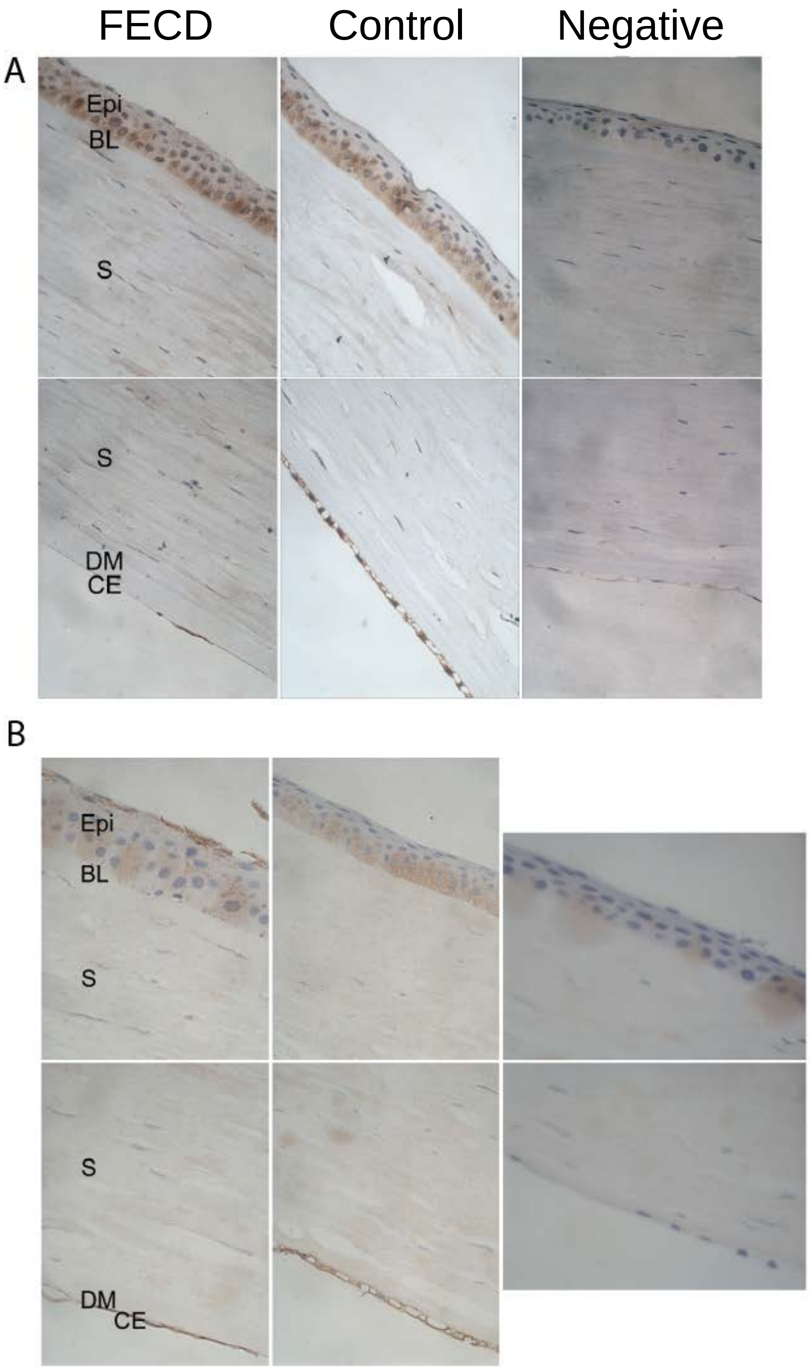

Each row of panels shows, left to right, FECD affected cornea, normal cornea and negative control with no primary antibody. Top and bottom panels show anterior and posterior halves of the cornea, respectively. Representative images from experiments in three independent pairs of corneas from FECD patients and controls are presented. **A.** Rat anti-human LAMC1 primary

antibody and goat anti-rat IgG secondary antibody. **B.** Mouse anti-human ATP1B1 primary antibody and rabbit anti-mouse IgG secondary antibody. All panels are at 400× magnification. Epi, epithelium; BL, Bowman layer; S, stroma; DM, Descemet's membrane; CE, corneal endothelium**.**

Supplementary Table 1. Phenotype criteria for FECD samples.

		Replication		
	Discovery	Iowa	Flinders	Johns Hopkins
Examination Method	Slit-lamp biomicroscopy	Slit-lamp biomicroscopy	Slit-lamp biomicroscopy	Slit-lamp biomicroscopy
Grading Scheme	Modified Krachmer[1]	Krachmer	Krachmer	Modified Krachmer
Minimum grade, FECD cases	3 in at least one eye, or keratoplasty	1 or histopathological confirmation	2	2
Minimum age, FECD controls, y	60	50	50	58
Genotyping	Illumina Omni2.5M (CIDR)	KASPer (LGC Genomics)	Sequenom iPLEX GOLD	TaqMan

FECD grading has been normalized to the modified Krachmer scale shown in Supplementary Table 2.

Supplementary Table 2. FECD grading scheme.

Grade	FECD Discovery	FECD Replication	Findings
0	Control	Control	No central cornea guttae
1	Not used	Not used	Scattered central cornea guttae
2	Not used	Case	1 or 2 mm of central cornea guttae
3	Case	Case	>2 to 5 mm of grouped central cornea guttae
4	Case	Case	>5 mm of grouped central cornea guttae
5	Case	Case	Cornea guttae with corneal edema

Grading scheme is based on the numerical scale of Krachmer et al.[1] and is equivalent to the scale of Louttit et al.[2] except that grades 1 and 2 of that scale are merged into grade 1.

Supplementary Table 3. Samples contributing to the discovery FECD GWAS.

	Severe FECD Cases					Controls		
Sample	*n*	Women	Age, y	FECD Grade	Histo.	*n*	Women	Age, y
FECD Multi-center	886	584 (65.9%)	69.9 ± 11.2*	4.03 ± 0.60	518 (58.5%)	526	304 (57.7%)	72.0 ± 8.2*
Duke U.	518	368 (71.0%)	69.4 ± 11.2	4.41 ± 1.04	270 (52.1%)	159	85 (53.5%)	70.5 ± 8.7
AREDS	0	NA	NA	NA	NA	1879	1107 (58.9%)	68.0 ± 4.7
**Total**	**1404**					**2564**		

Age and FECD Grade are shown as mean ± SD; Histo., number (%) of cases confirmed to have severe FECD by histopathological examination. *, significantly different between cases and controls ($p < 0.001$ by Student's t test for age or Pearson's χ^2 test for percentage women).

Supplementary Table 4. Most significant association results in previously reported FECD candidate genes.

Chr.	Gene	No. SNPs	Top SNP	Position	Alleles	EAF Cases	EAF Ctrls	OR	95% CI	*p*
1	*COL8A2*	94	rs274751	36,562,078	C/T	0.0089	0.0098	1.78	(1.05, 3.02)	0.031
3	*COL8A1*	452	rs184124635	99,368,636	T/C	0.0080	0.0125	0.51	(0.29, 0.91)	0.023
3	*PTPRG*	2554	rs178331951	61,812,765	T/G	0.0139	0.0080	2.11	(1.28, 3.45)	0.0032
10	*TCF8*	366	rs182661226	31,737,986	T/C	0.0207	0.0141	1.68	(1.12, 2.52)	0.012
15	*AGBL1*	3934	rs118086539	86,769,958	T/C	0.0192	0.0086	2.28	(1.52, 3.43)	7.53E-05
18	*LOXHD1*	682	rs450997	44,244,840	G/A	0.6664	0.7012	0.83	(0.75, 0.92)	0.00034
20	*SLC4A11*	120	rs140676642	3,220,190	T/G	0.0266	0.0204	1.46	(1.03, 2.05)	0.031

Chr., chromosome; No. SNPs, number of markers in and within 10 kilobasepairs (kb) of the gene; Top SNP, marker with smallest *p* value within 10 kb of gene; Alleles, effect/other allele. *COL8A2* [3,4] has been implicated in early-onset FECD, whereas *PTPRG* [5], *TCF8* (*ZEB1*) [6,7], *AGBL1* [8], *LOXHD1* [9] and *SLC4A11* [10-12] have been associated with late-onset FECD. *COL8A1* was nominated as a candidate FECD risk locus through its expression in Descemet's membrane [13], but no mutations in this gene have been observed in FECD patients [14,15].

Supplementary Table 5. Samples contributing to the FECD replication cohort.

	FECD Cases			Controls		
Sample	*n*	Female	Age, y	*n*	Female	Age, y
U. of Iowa	113	79 (69.9%)	72 ± 14	113	64 (56.6%)	71 ± 9
Hopkins	368	194 (52.7%)	57 ± 7.6	380	202 (53.2%)	69 ± 2.6
Flinders U.	190	131 (68.9%)	70 ± 11	282	158 (56.0%)	76 ± 8.2
**Total**	**671**			**778**		

Female, number (%) of women; Age and FECD grade are shown as mean ± SD.

Supplementary Table 6. Association with FECD in discovery and replication cohorts in all SNPs tested for replication.

					Discovery Cohort				Replication Cohorts		Combined		
SNP	Chr	Pos	Gene	All.	RAF Case	RAF Ctrl	OR (95% CI)	p_{disc}	OR (95% CI)	p_{repl}	OR (95% CI)	$p_{combined}$	Q *p*
rs12082238	1	62760625	*KANK4*	C/T	0.2366	0.2896	0.75 (0.67, 0.83)	1.8E-07	0.81 (0.69, 0.97)	1.9E-02	0.76 (0.70, 0.84)	1.5E-08	0.46
rs12058486*	1	62767265	*KANK4*	T/C	0.2297	0.2856	0.73 (0.65, 0.82)	3.4E-08	0.89 (0.75, 1.06)	2.0E-01	0.78 (0.71, 0.85)	1.0E-07	0.20
rs79742895*	1	62782860	*KANK4*	C/T	0.0889	0.0474	2.07 (1.69, 2.54)	2.5E-12	2.18 (1.36, 3.50)	1.2E-03	2.09 (1.73, 2.52)	1.2E-14	0.83
rs1200114	1	169060489	*ATP1B1*	G/A	0.4277	0.3411	1.43 (1.29, 1.57)	8.0E-13	1.53 (1.30, 1.79)	1.7E-07	1.45 (1.34, 1.58)	9.9E-19	0.48
rs1200115	1	169061558	*ATP1B1*	T/C	0.3974	0.3261	1.35 (1.23, 1.49)	1.7E-09	1.49 (1.26, 1.75)	1.5E-06	1.39 (1.28, 1.51)	2.1E-14	0.46
rs2296292*[†]	1	183086757	*LAMC1*	A/C	0.5075	0.4503	1.27 (1.15, 1.39)	1.2E-06	1.40 (1.20, 1.63)	1.5E-05	1.30 (1.20, 1.41)	1.5E-10	0.69
rs3768617*	1	183092500	*LAMC1*	A/G	0.3553	0.4214	0.75 (0.68, 0.82)	8.8E-09	0.62 (0.53, 0.72)	2.1E-09	0.71 (0.65, 0.77)	6.9E-16	0.26
rs1413386*	1	183104811	*LAMC1*	C/G	0.3792	0.4456	0.75 (0.68, 0.83)	1.6E-08	0.74 (0.63, 0.86)	1.0E-04	0.75 (0.69, 0.81)	7.1E-12	0.57
rs20560*[†]	1	183105534	*LAMC1*	T/C	0.511	0.4528	1.27 (1.15, 1.40)	9.2E-07	1.46 (1.17, 1.81)	6.9E-04	1.30 (1.19, 1.42)	4.6E-09	0.50
rs20561	1	183105705	*LAMC1*	A/G	0.3553	0.4189	0.76 (0.69, 0.84)	5.4E-08	0.64 (0.53, 0.78)	5.2E-06	0.73 (0.67, 0.80)	4.5E-12	0.25
rs2274700	1	196682947	*CFH*	T/C	0.4287	0.3709	1.31 (1.19, 1.44)	3.4E-08	1.08 (0.91, 1.27)	3.8E-01	1.25 (1.15, 1.36)	1.8E-07	0.097
rs1329428	1	196702810	*CFH*	A/G	0.4287	0.3707	1.31 (1.19, 1.44)	3.2E-08	1.04 (0.90, 1.22)	5.8E-01	1.23 (1.13, 1.33)	6.4E-07	0.062
rs12223324	11	772701	*SLC25A22*	A/G	0.4826	0.5445	0.77 (0.70, 0.85)	2.1E-07	0.92 (0.79, 1.07)	2.7E-01	0.81 (0.75, 0.88)	7.3E-07	0.24
rs4963153	11	791462	*SLC25A22*	T/C	0.4736	0.5437	0.74 (0.67, 0.82)	2.0E-09	1.06 (0.91, 1.24)	4.1E-01	0.82 (0.76, 0.89)	4.0E-06	0.00064
rs1138714s	11	825110	*SLC25A22*	A/G	0.4598	0.3959	0.76 (0.69, 0.84)	3.4E-08	0.97 (0.84, 1.13)	7.4E-01	0.82 (0.75, 0.89)	1.5E-06	0.056
rs72932713*	18	53186911	*TCF4*	C/T	0.2859	0.0549	9.76 (8.22, 11.59)	8.4E-149	5.29 (3.72, 7.52)	1.8E-20	8.67 (7.43, 10.12)	1.8E-165	0.0034
rs613872*	18	53210302	*TCF4*	G/T	0.4743	0.1640	6.44 (5.62, 7.39)	6.4E-156	4.23 (3.13, 5.71)	2.1E-22	5.99 (5.29, 6.78)	6.6E-174	0.012
rs11659764*	18	53335512	*TCF4*	A/T	0.2915	0.0516	10.78 (9.06, 12.83)	2.3E-157	6.34 (4.36, 9.21)	3.4E-22	9.80 (8.37, 11.48)	2.2E-176	0.022
rs784257	18	53397199	*TCF4*	A/G	0.4779	0.1796	5.77 (5.05, 6.60)	3.1E-146	3.89 (3.30, 4.59)	6.3E-59	4.94 (4.45, 5.48)	2.5E-200	0.0041

Chr, chromosome; Pos, build GRCh37 map position; All., reference/other alleles; RAF Case, RAF Ctrl., reference allele frequencies in FECD cases and controls; p_{disc}, p_{repl}, $p_{combined}$, p value in discovery, replication and combined cohorts, respectively; Q.p, p value from Cochran's Q test for heterogeneity; *imputed in discovery data set; †coding variant

Supplementary Table 7. Power analysis for the discovery GWAS.

	Effect Allele Frequency									
Allele RR	0.01	0.02	0.03	0.04	0.05	0.10	0.15	0.20	0.30	0.40
1.2	0.0000	0.0000	0.0000	0.0001	0.0001	0.0012	0.0048	0.0118	0.0334	0.0527
1.3	0.0000	0.0001	0.0003	0.0009	0.0021	0.0273	0.1000	0.2078	0.4157	0.5289[a]
1.4	0.0000	0.0005	0.0025	0.0078	0.0185	0.1938	0.4827	0.7088	**0.9031**[b]	**0.9494**[a]
1.5	0.0002	0.0026	0.0134	0.0407	0.0907	0.5575	**0.8710**	**0.9669**	**0.9967**	**0.9990**
1.6	0.0006	0.0102	0.0499	0.1374	0.2685	**0.8659**	**0.9877**	**0.9989**	**0.9999**	**0.9999**
1.8	0.0049	0.0766	0.2848	0.5540	0.7700	**0.9982**	**0.9999**	**0.9999**	**0.9999**	**0.9999**
2.0	0.0236	0.2755	0.6685	**0.8995**	**0.9772**[c]	**0.9999**	**0.9999**	**0.9999**	**0.9999**	**0.9999**
2.2	0.0776	0.5735	**0.9157**	**0.9905**	**0.9992**	**0.9999**	**0.9999**	**0.9999**	**0.9999**	**0.9999**
2.4	0.1866	**0.8217**	**0.9882**	**0.9996**	**0.9999**	**0.9999**	**0.9999**	**0.9999**[d]	**0.9999**	**0.9999**

The assumed mode of inheritance was log additive with a population prevalence for FECD of 0.04, a nominal detection *p* value of 5e–08, and a sample size equal to that of the discovery sample. Power values above 0.8 are in **boldface.** The models closest to the observed modes of inheritance are indicated by letter for the most significantly associated SNPs in [a]*LAMC1*, [b]*ATP1B1*, [c]*KANK4* and [d]*TCF4*. The *LAMC1* observed model is intermediate between the two models marked *a*.

Supplementary Table 8. Proportion of variation in FECD in the discovery sample explained by most significant markers in replicated association peaks.

Gene	Marker	OR	RAF	%Var
KANK4	rs79742895*	2.07	0.062	1.48
ATP1B1	rs1200114	1.43	0.372	1.27
LAMC1	rs3768617*	1.34	0.602	0.86
TCF4	rs784257	5.78	0.285	21.89

Proportion of variation explained was calculated using the liability model of So et al.[16] for the most strongly associated SNP within each association peak from the Illumina Omni2.5 or 1000 Genomes imputed GWAS. RAF, risk allele frequency; %Var, percentage of variance of FECD explained by marker under a multiplicative odds model; *, imputed marker.

Supplementary Table 9. Power analysis for SNP × SNP interaction effect when one SNP is rs784257 in *TCF4*.

	Gene	KANK4	ATP1B1	LAMC1
	SNP	rs79742895	rs2100114	rs3768617
	EAF	0.0474	0.332	0.438
	Main RR	2.07	1.43	0.75
Power,	1.2	0.1612	0.4680	0.4908
Interaction RR	1.3	0.2812	0.7545	0.7804
	1.4	0.4211	**0.9142**	**0.9298**
	1.6	0.6828	**0.9939**	**0.9958**
	1.8	**0.8550**	**0.9997**	**0.9998**
	2.0	**0.9416**	**0.9999**	**0.9999**

One marker was assumed to have allelic RR = 5.77 and EAF = 0.187 (from 1000 Genomes EUR samples); the mode of inheritance for the second SNP was as shown. Power values above 0.8 are in **boldface.**

Supplementary Table 10. Results from association analysis conditioning on *TCF4* SNP rs784257.

		Unconditioned		Conditioned	
Gene	Marker	OR (95% CI)	*p*	OR (95% CI)	*p*
KANK4	rs79742895*	2.07 (1.69, 2.54)	2.5E-12	1.99 (1.58, 2.51)	7.1E-09
ATP1B1	rs1200114	1.43 (1.29, 1.57)	8.0E-13	1.47 (1.31, 1.64)	1.5E-11
LAMC1	rs3768617*	0.75 (0.68, 0.82)	8.8E-09	0.77 (0.69, 0.86)	7.5E-06

Unconditioned, same as in Supplementary Table 3; Conditioned, result from logistic regression in which the number of risk alleles at rs784257 was included as a predictor; *, imputed marker.

Supplementary Table 11. Comparison of FECD GWAS cases within the discovery cohort with and without histopathological examination.

	Verified (*n* = 787)	Other Cases (616)	*p*
Female	529 (67.3%)	423 (68.7%)	0.58
Age, y	70.9 (10.2)	68.2 (12.2)	< 0.001
FECD Grade	4.40 (0.50)	3.83 (0.99)	< 0.001

Female, number (%) of women; Age and FECD Grade are shown as mean ± SD. *p* value is from 2 × 2 χ^2 test (Female) or from Student's *t* test (Age, FECD Grade).

Supplementary Table 12. Results from association analysis limiting to FECD cases with FECD confirmed by histopathology.

		All FECD Cases (n = 1404)		Histopathology Only (n = 788)	
Gene	Marker	OR (95% CI)	p	OR (95% CI)	p
KANK4	rs79742895*	2.07 (1.69, 2.54)	2.5E-12	2.23 (1.76, 2.84)	6.1E-11
ATP1B1	rs1200114	1.43 (1.29, 1.57)	8.0E-13	1.49 (1.32, 1.68)	9.7E-11
LAMC1	rs3768617*	0.75 (0.68, 0.82)	8.8E-09	0.73 (0.64, 0.82)	4.7E-07
CFH	rs2274700	1.31 (1.19, 1.44)	3.4E-08	1.29 (1.14, 1.45)	2.89E-05
SLC25A22	rs12223324	0.77 (0.70, 0.85)	2.1E-07	0.76 (0.67, 0.86)	9.67E-06
TCF4	rs784257	5.77 (5.05, 6.60)	3.1E-146	6.38 (5.42, 7.52)	2.01E-109

All FECD Cases, same as in Supplementary Table 3, discovery cohort; Histopathology Only, FECD cases without histopathologic confirmation omitted; *, imputed marker. Both samples were compared to 2,564 FECD controls from the discovery cohort.

Supplementary Table 13. Sex-stratified subset association analysis and heterogeneity test.

			Men Only		Women Only		
Gene	Marker	Ref. Allele	OR (95% CI)	*p*	OR (95% CI)	*p*	*p* Het.
KANK4	rs79742895*	C	2.31 (1.62, 3.30)	3.7E-06	2.17 (1.66, 2.83)	1.0E-08	0.78
ATP1B1	rs1200114	G	1.48 (1.26, 1.75)	2.5E-06	1.39 (1.23, 1.57)	1.23E-07	0.55
LAMC1	rs3768617*	C	1.16 (0.98, 1.34)	9.2E-02	1.51 (1.32, 1.72)	6.9E-10	*0.017*
CFH	rs2274700	A	1.18 (1.00, 1.39)	4.9E-02	1.39 (1.24, 1.57)	5.36E-08	0.11
SLC25A22	rs12223324	A	0.69 (0.58, 0.82)	1.3E-05	0.81 (0.72, 0.92)	7.2E-04	0.13
TCF4	rs784257	A	7.56 (5.96, 9.57)	4.7E-63	5.06 (4.29, 5.96)	1.8E-83	**0.0063**

Men and Women Only, results from association analysis on FECD using only male and female samples from the discovery cohort, respectively; *p* Het, *p* value from the χ^2 test of heterogeneity[17] with *p* values between 0.01 and 0.05 in italics and *p* values below 0.01 in boldface; *, imputed marker not in the Omni2.5 panel, for which allele dosage data were used.

Supplementary Table 14. Power analysis for gene × sex interaction effect for observed main-effect models of rs3768617 in *LAMC1* and rs784257 in *TCF4*.

rs3768617, LAMC1

	Effect Allele Frequency						
Interaction RR	0.05	0.10	0.15	0.20	0.25	0.30	0.40
1.2	0.1389	0.2200	0.2900	0.3482	0.3949	0.4309	0.4745
1.4	0.3673	0.6009	0.7451	**0.8300**	**0.8796**	**0.9088**	**0.9348**
1.6	0.6303	**0.8777**	**0.9730**	**0.9830**	**0.9921**	**0.9956**	**0.9977**
1.8	**0.8252**	**0.9757**	**0.9960**	**0.9992**	**0.9998**	**0.9999**	**0.9999**
2.0	**0.9306**	**0.9965**	**0.9998**	**0.9999**	**0.9999**	**0.9999**	**0.9999**

Power to detect a nominal interaction *p* value of 0.05 is indicated given population parameters: FECD prevalence of 0.4 and equal proportions of men and women. Values above 0.8 are in **boldface.** Power for the observed mode of inheritance (EAF = 0.438 in 1000 Genomes EUR samples, parameters indicated in Supplementary Table 10) was 0.7668.

rs784257, TCF4

	Effect Allele Frequency						
Interaction RR	0.05	0.10	0.15	0.20	0.25	0.30	0.40
1.2	0.1786	0.2374	0.2535	0.2571	0.2601	0.2634	0.2608
1.4	0.4757	0.6187	0.6454	0.6474	0.6518	0.6582	0.6503
1.6	0.7515	**0.8765**	**0.8898**	**0.8878**	**0.8895**	**0.8934**	**0.8851**
1.8	**0.9073**	**0.9706**	**0.9738**	**0.9717**	**0.9718**	**0.9731**	**0.9680**
2.0	**0.9709**	**0.9942**	**0.9946**	**0.9935**	**0.9934**	**0.9937**	**0.9913**

Population parameters are the same as in the previous table. Values above 0.8 are in **boldface.** Power for the observed mode of inheritance (EAF = 0.187 in 1000 Genomes EUR samples, parameters indicated in Supplementary Table 10) was 0.8707.

Supplementary References

1. Krachmer, J.H., Purcell, J.J., Jr., Young, C.W. & Bucher, K.D. Corneal endothelial dystrophy. A study of 64 families. *Arch. Ophthalmol.* **96**, 2036-2039 (1978).
2. Louttit, M.D. *et al.* A multi-center study to map genes for Fuchs' endothelial corneal dystrophy: baseline characteristics and heritability. *Cornea* **31**, 26-35 (2012).
3. Biswas, S. *et al.* Missense mutations in *COL8A2,* the gene encoding the α2 chain of type VIII collagen, cause two forms of corneal endothelial dystrophy. *Hum. Mol. Genet.* **10**, 2415-2423 (2001).
4. Gottsch, J.D. *et al.* Inheritance of a novel *COL8A2* mutation defines a distinct early-onset subtype of Fuchs corneal dystrophy. *Invest. Ophthalmol. Vis. Sci.* **46**, 1934-1939 (2005).
5. Baratz, K.H. *et al.* E2-2 protein and Fuchs's corneal dystrophy. *N. Engl. J. Med.* **363**, 1016-1024 (2010).
6. Mehta, J.S. *et al.* Analysis of the posterior polymorphous corneal dystrophy 3 gene, *TCF8,* in late-onset Fuchs endothelial corneal dystrophy. *Invest. Ophthalmol. Vis. Sci.* **49**, 184-188 (2008).
7. Riazuddin, S.A. *et al.* Missense mutations in *TCF8* cause late-onset Fuchs corneal dystrophy and interact with *FCD4* on chromosome 9p. *Am. J. Hum. Genet.* **86**, 45-53 (2010).
8. Riazuddin, S.A., Vasanth, S., Katsanis, N. & Gottsch, J.D. Mutations in *AGBL1* cause dominant late-onset Fuchs corneal dystrophy and alter protein-protein interaction with TCF4. *Am. J. Hum. Genet.* **93**, 758-764 (2013).
9. Riazuddin, S.A. *et al.* Mutations in *LOXHD1*, a recessive-deafness locus, cause dominant late-onset Fuchs corneal dystrophy. *Am. J. Hum. Genet.* **90**, 533-539 (2012).
10. Vithana, E.N. *et al.* Mutations in sodium-borate cotransporter *SLC4A11* cause recessive congenital hereditary endothelial dystrophy (CHED2). *Nat. Genet.* **38**, 755-757 (2006).
11. Desir, J. *et al.* Borate transporter *SLC4A11* mutations cause both Horboyan syndrome and non-syndromic corneal endothelial dystrophy. *J. Med. Genet.* **44**, 322-326 (2007).

12. Riazuddin, S.A. *et al.* Missense mutations in the sodium borate cotransporter *SLC4A11* cause late-onset Fuchs corneal dystrophy. *Hum. Mutat.* **31**, 1261-1268 (2010).

13. Kapoor, R. *et al.* Type VIII collagen has a restricted distribution in specialized extracellular matrices. *Journal of Cell Biology* **107**, 721-730 (1988).

14. Aldave, A.J. *et al.* No pathogenic mutations identified in the *COL8A1* and *COL8A2* genes in familial Fuchs corneal dystrophy. *Invest. Ophthalmol. Vis. Sci.* **47**, 3787-3790 (2006).

15. Urquhart, J.E., Biswas, S., Black, G.C.M., Munier, F.L. & Sutphin, J. Exclusion of *COL8A1*, the gene encoding the a_2(VIII) chain of type VIII collagen, as a candidate for Fuchs endothelial dystrophy and posterior polymorphous corneal dystrophy. *Brit. J. Ophthalmol.* **90**, 1430-1431 (2006).

16. So, H.-C., Gui, A.H.S., Cherny, S.S. & Sham, P.C. Evaluation the heritability explained by known susceptibility variants: a survey of ten complex diseases. *Genet. Epidemiol.* **35**, 310-317 (2011).

17. Mägi, R., Lidgren, C.M. & Morris, A.P. Meta-analysis of sex-specific genome-wide association studies. *Genet. Epidemiol.* **34**, 846-853 (2010).
